# CSPG4 as a target for the specific killing of triple-negative breast cancer cells by a recombinant SNAP-tag-based antibody-auristatin F drug conjugate

**DOI:** 10.1007/s00432-023-05031-3

**Published:** 2023-07-11

**Authors:** Neelakshi Mungra, Fleury A. N. Biteghe, Zaria Malindi, Allan M. Huysamen, Maryam Karaan, Natasha S. Hardcastle, Rubina Bunjun, Shivan Chetty, Krupa Naran, Dirk Lang, Wolfgang Richter, Roger Hunter, Stefan Barth

**Affiliations:** 1 Institute of Infectious Disease and Molecular Medicine, Medical Biotechnology and Immunotherapy Research Unit, University of Cape Town, Cape Town, 7700 South Africa; 2 Centre for Immunity and Immunotherapies, Seattle Children’s Research Institute, Washington, 98101 USA; 3 Department of Radiation Oncology and Biomedical Sciences, Cedars-Sinai Medical, Los Angeles, USA; 4 Faculty of Health Sciences, Laser Research Centre, University of Johannesburg, Doornfontein, Johannesburg, 2028 South Africa; 5 Department of Chemistry, PD Hahn Building, University of Cape Town, Cape Town, 7700 South Africa; 6 Institute of Infectious Disease and Molecular Medicine, University of Cape Town, Cape Town, 7700 South Africa; 7 Division of Medical Virology, Department of Pathology, University of Cape Town, Cape Town, 7700 South Africa; 8 Faculty of Health Sciences, School of Clinical Medicine, University of Witwatersrand, Braamfontein, Johannesburg, 2000 South Africa; 9 Division of Physiological Sciences, Department of Human Biology, University of Cape Town, Cape Town, 7700 South Africa; 10 TUBE Pharmaceuticals, 1110 Vienna, Austria; 11 Faculty of Health Sciences, Department of Integrative Biomedical Sciences, South African Research Chair in Cancer Biotechnology, University of Cape Town, Cape Town, 7700 South Africa

**Keywords:** Triple-negative breast cancer (TNBC), Chondroitin sulfate proteoglycan 4 (CSPG4), Click chemistry for single-chain antibody fragment (scFv), Recombinant antibody–drug conjugate (ADC), SNAP-tag based fusion protein, Benzylguanine (BG)-modified auristatin F (AURIF)

## Abstract

**Purpose:**

Triple-negative breast cancer (TNBC) is phenotypic of breast tumors lacking expression of the estrogen receptor (ER), the progesterone receptor (PgR), and the human epidermal growth factor receptor 2 (HER2). The paucity of well-defined molecular targets in TNBC, coupled with the increasing burden of breast cancer-related mortality, emphasizes the need to develop targeted diagnostics and therapeutics. While antibody–drug conjugates (ADCs) have emerged as revolutionary tools in the selective delivery of drugs to malignant cells, their widespread clinical use has been hampered by traditional strategies which often give rise to heterogeneous mixtures of ADC products.

**Methods:**

Utilizing SNAP-tag technology as a cutting-edge site-specific conjugation method, a chondroitin sulfate proteoglycan 4 (CSPG4)-targeting ADC was engineered, encompassing a single-chain antibody fragment (scFv) conjugated to auristatin F (AURIF) via a click chemistry strategy.

**Results:**

After showcasing the self-labeling potential of the SNAP-tag component, surface binding and internalization of the fluorescently labeled product were demonstrated on CSPG4-positive TNBC cell lines through confocal microscopy and flow cytometry. The cell-killing ability of the novel AURIF-based recombinant ADC was illustrated by the induction of a 50% reduction in cell viability at nanomolar to micromolar concentrations on target cell lines.

**Conclusion:**

This research underscores the applicability of SNAP-tag in the unambiguous generation of homogeneous and pharmaceutically relevant immunoconjugates that could potentially be instrumental in the management of a daunting disease like TNBC.

**Supplementary Information:**

The online version contains supplementary material available at 10.1007/s00432-023-05031-3.

## Introduction

According to the World Health Organization, 2.3 million women were diagnosed with breast cancer and 685 000 women died from breast cancer worldwide in 2020 (World Health Organization 2021), making it the most commonly diagnosed cancer and the most frequent cause of cancer-related deaths in most regions across the globe (Ferlay et al. 2021). Breast tumors are phenotypically diverse and are thus classified into five subtypes based on the corresponding gene expression patterns of epidermal growth factor receptor (EGFR) 2 (known as HER2/neu) (which is overexpressed in approximately 20% of all breast tumors), estrogen receptor-α (ER), and progesterone receptor (PgR) (Zhiyuan Hu et al. 2006; Sørlie et al. 2001). These molecular subtypes are namely luminal A, luminal B, HER2-positive, basal-like, and normal breast-like subtypes. The normal breast-like subtype is also known as triple-negative breast cancer (TNBC), because it lacks expression of HER2, ER, and PgR; however, unlike the basal-like subtype, which similarly lacks these three hormone receptors, TNBC is also negative for keratin 5 (CK5) and EGFR expression (Yersal and Barutca 2014).

TNBC accounts for up to 20% of all breast cancer cases, and compared to other subtypes that are positive for hormone receptor expression, it is typically associated with more aggressive progression and poorer treatment outcomes (Zhao et al. 2020). To date, only one antibody–drug conjugate (ADC) has been approved for TNBC; sacituzumab govitecan, which targets human trophoblast cell-surface antigen 2 (Wahby et al. 2021). TNBC is in itself a heterogeneous disease, and as such, it exhibits subpar chemotherapy responses with limited alternative treatment options due to the absence of appropriate therapeutic molecular targets (Zhao et al. 2020). This is contrary to hormone receptor-positive patients who typically show acceptable clinical responses to common endocrine therapies and monoclonal antibodies (mAbs) (Korde et al. 2021).

Over the last few decades, site-directed drug delivery has become increasingly popular in the form of ADCs that can selectively bind tumor-associated antigens (TAAs) or tumor-specific antigens. This form of cancer treatment harnesses the precision of antibody-based medicine while allowing for the delivery of potent drugs such as auristatin F (AURIF), that might otherwise be too toxic as free drugs or might act non-specifically, causing systemic and dose-limiting side effects even at low concentrations (Fitting et al. 2015; Nasiri et al. 2018). ADCs can additionally be considered superior to mAb therapies, because they are not reliant on the immune responses of a compromised host system but rather offer greater and faster-acting cytotoxic potency, thus increasing the chances of effective cancer cell destruction (Chen and Mellman 2017; Strebhardt and Ullrich 2008). Nonetheless, ADC technologies have, by necessity, advanced since the first ADC trial conducted in 1983 (Ford et al. 1983). Earlier generations of ADCs were formulated utilizing the conventional conjugation methods that required cross-linking native cysteine or lysine amino acid side chains; however, this technique led to heterogeneous conjugate production (McCombs and Owen 2015). Inconsistent drug-to-antibody ratios (DARs) associated with non-specific conjugation techniques affect the pharmacokinetics and resultant efficacy of these conjugate drug batches, creating difficulties regarding the reproducibility of individual batches (Harris and Chess 2003).

As such, improved conjugation methods have been developed to overcome the challenges of early ADCs. With the FDA approval of 12 ADCs as of January 2023 (Coleman et al. 2023), most of which have occurred within the last 5 years (Hueper 2021), research in this area is generating much interest and momentum. Interestingly, SNAP-tag has presented a promising technology for antibody engineering and ADC development. Derived from the O6-alkylguanine-DNA-alkyltransferase (hAGT) human DNA repair enzyme, SNAP-tag exhibits directed and autocatalytic reactivity with benzylguanine (BG)-modified substrates (Dolan et al. 1990; Keppler et al. 2004). Covalent bonding between SNAP-tag and any BG derivative occurs at a defined binding site in a predictable 1:1 vehicle-to-payload ratio to produce a homogeneous conjugation product via an irreversible process (Pegg et al. 1995). By generating fusion proteins comprising SNAP-tag and an antibody fragment, the conjugation properties of SNAP-tag can be endowed onto the antibody, allowing for rapid and efficient self-labeling of the antibody with the appropriate BG-modified substrate (Kampmeier et al. 2009, 2010; Hussain et al. 2013).

One molecular target that has been identified for its potential use in TNBC is chondroitin sulfate proteoglycan 4 (CSPG4). CSPG4 plays an important role in cell migration and survival pathways and has been shown to be overexpressed in TNBC cells. Moreover, its inhibition using CSPG4-specific mAbs has been shown to significantly reduce the tumorigenic power of TNBC cells and mitigate metastases and recurrence in xenograft mouse models (Yang et al. 2004; Uranowska et al. 2021; Wang et al. 2010a, b). CSPG4 exhibits minimal expression in healthy, mature adult tissues and thus offers an ideal target for site-directed drug delivery to selected cells with minimal off-target accumulation that would otherwise result in systemic side effects (Yang et al. 2009; Natali et al. 1985; Ghosh et al. 2020). Moreover, being membrane-bound, CSPG4 undergoes negligible secretion into the bloodstream, which makes it suitable as a target, since ADC-antigen binding in circulation will not interfere with the targeting precision of the therapy (Wang et al. 2010a, b; Hoffmann et al. 2020).

In this study, the overarching aim was to develop an ADC product targeting CSPG4-positive TNBC cells in vitro. Therefore, the first step involved the engineering of a recombinant antibody single-chain variable fragment (scFv) against CSPG4 in the form of a SNAP-tag-based fusion protein. Next, BG-modified AURIF was synthesized by employing a click chemistry coupling strategy, whereby an AURIF–linker–BG payload was created for SNAP-tag coupling (Huysamen et al. 2023). Thereafter, the binding selectivity and internalization of the fusion protein was verified, and the selective in vitro cytotoxicity of the conjugate was evaluated.

## Materials and methods

### Cell culture

Human embryonic kidney cells (HEK293T) (ATCC: CRL-11268), the human breast carcinoma cell MCF-7 (ATCC: HTB-22), and the human melanoma cell line SK-Mel-28 (ATCC: HTB-72) were cultured in RPMI-1640 medium (containing 2 mM l-glutamine, 3.7 g/L NaHCO_3_ and 15 mg/L phenol red), supplemented with 10% (v/v) heat-inactivated fetal bovine serum (FBS) and 1% (v/v) 100 U/mL penicillin–streptomycin. Similarly, the human TNBC cell lines, Hs578T (ATCC: HTB-126), MDA-MB-468 (ATCC: HTB-132), and MDA-MB-231 (ATCC: HTB-26) were all cultured in Dulbecco’s Modified Eagle Medium (DMEM) (containing 2 mM l-glutamine, 3.7 g/L C_3_H_3_NaO_3_ and 16 mg/L phenol red), supplemented with 10% (v/v) heat-inactivated FBS and 1% (v/v) 100 U/mL penicillin–streptomycin. The cells were maintained in a 5% CO_2_ incubator with 95% humidity at 37 °C. Medium was changed every 3–4 days and cells were passaged when 90% confluent. All products were purchased from Gibco by Life Technologies^®^ (RPMI-1640: Gibco #10566 and DMEM: Gibco #61870), supplied by Thermo Fisher Scientific, South Africa.

### Molecular cloning and expression of αCSPG4(scFv)-SNAP

To engineer a CSPG4-targeting scFv, the variable heavy chain (*V*_H_) was linked to its corresponding variable light chain (*V*_L_) (note: the *V*_H_*V*_L_ gene sequences were extracted from the CSPG4-specific mAb9.2.27 and combined into an scFv format as described by Schwenkert et al. 2008), and the resulting complementarity-determining region (CDR) was then aligned to its parental sequence using CLC genomic workbench v12 software. The newly designed scFv sequence was then inserted into the prototype pCB-H22(scFv)-SNAP expression plasmid (previously generated at MB&I) between *Sfi*I and *Not*I restriction sites, using SnapGene software (version 3.1.1, GSL Biotech, Chicago). After confirming the integrity of the open-reading frame (ORF) in silico, the scFv sequence was cloned into the plasmid expression vector, resulting in the generation of pCB-αCSPG4(scFv)-SNAP (pCB-mAb9.2.27(scFv)-SNAP).

Upon confirmation of the resulting DNA sequences [via Sanger sequencing using an ABI 3500XL genetic analyzer (Inqaba Biotec™, South Africa)], the eukaryotic expression vector system (1 µg/µL) was transiently transfected into HEK293T cells (at 70–80% confluency) using XtremeGene™ transfection reagent (Sigma-Aldrich, South Africa) according to the manufacturer’s instructions. This procedure utilizes lipids and polymers that are capable of complexing with DNA to form micelles, which in turn facilitate the uptake of DNA into mammalian cells. A 1:3 ratio of DNA-to-transfection reagent was used (3 µL of DNA and 9 µL of transfection reagent) and mixed with 188 µL of serum-free and antibiotic-free RPMI-1640 (Gibco #10566, containing 2 mM l-glutamine, 3.7 g/L NaHCO_3_, and 15 mg/L phenol red). Untransfected HEK293T cells were included as a negative control. The transfected cells were then grown in RPMI-1640 culture medium (Gibco #10566) supplemented with 10% (v/v) FBS and 1% (v/v) 100 U/mL penicillin–streptomycin. Plasmid uptake was then assessed after 3 days, through the microscopic visualization of enhanced green fluorescent protein (eGFP) expressed by the transfected cells (as an indication of successful transfection and potential transient expression of the putative fusion protein), using a ZOE™ Fluorescent Cell Imager (Bio-Rad Laboratories, UK). To determine the transfection efficiency, 2 mL of the transfected cells and controls were subjected to flow cytometry using the BD™ LSR II flow cytometer (BD Biosciences, USA). Transfection efficiency was expressed as a percentage of the eGFP-positive cells present within the total population. Zeocin selection (100 µg/mL) was then applied to enrich the eGFP-positive cells containing the recombinant plasmids. These cells were then grown at 90% confluency and the cell culture supernatant containing the secreted protein of interest was harvested every 4 days, for a period of ± 6 months or until sufficient protein (> 1 mg/mL) was obtained. The collected supernatant was pooled, centrifuged at 2500 rpm for 3 min to remove cellular debris, and then stored at 4 °C until protein purification.

### Protein purification using immobilized metal affinity chromatography (IMAC)

The cell culture supernatant [1 part of cell culture supernatant was mixed with 3 parts of 4 × incubation buffer (200 mM NaH_2_PO_4_, 1.2 M NaCl, 40 mM imidazole, pH 8.0) to ensure optimal binding conditions] of αCSPG4(scFv)-SNAP was first filtered using the Nalgene™ vacuum filtration system (Sigma-Aldrich, South Africa) containing a 0.45 μm Durapore^®^ membrane filter (Millipore, USA) to exclude any microcellular debris, before purification by Immobilized Metal Affinity Chromatography (IMAC). IMAC was carried out using a Ni^2+^ affinity resin (packed in a HisTrap™ Excel column, GE Healthcare, USA) on an ÄKTA Avant protein purification system (GE Healthcare, USA). Initially, each clarified cell culture supernatant was applied on a pre-equilibrated HisTrap™ Excel column at a flow rate of 5 mL/min. Thereafter, the column was washed with 20 column volumes of equilibration buffer (50 mM NaH_2_PO_4_, 300 mM NaCl, pH 8.0) and the bound fusion proteins were eluted using elution buffer (50 mM NaH_2_PO_4_, 300 mM NaCl, 500 mM imidazole, pH 8.0) containing a high concentration of imidazole. In principle, fractional elution of the his-tagged fusion proteins is made possible by increasing the concentration of imidazole, which competes with histidine for binding to the metal-charged resin. Thereafter, to concentrate the eluted fractions (as well as remove residual imidazole), 10 K-sized Amicon filters (Sigma-Aldrich, South Africa) were used. The samples were centrifuged at 4500×*g* for 20 min at 4 °C and washed in 1 × phosphate-buffered saline (pH 7.4) (henceforth referred to as 1 × PBS, containing 137 mM NaCl, 8.8 mM Na_2_HPO_4_, 2.7 mM KCl, and 1.75 mM KH_2_PO_4_) thrice prior to downstream assays (note: 1 × PBS was also used as the protein storage buffer at − 20 °C). Protein quantification was assessed by UV spectrophotometry, using a DeNovix DS-11 (DeNovix, USA), prior to further characterization.

### Sodium dodecyl sulfate–polyacrylamide gel electrophoresis (SDS-PAGE) and western blot analysis of recombinant protein fractions

A discontinuous 10% SDS-PAGE gel was used to resolve proteins electrophoretically on the basis of their molecular weights. For sample preparation, 15 µL of the recombinant protein samples was mixed with 5 µL of 4 × Laemmli protein sample buffer (Bio-Rad, USA) supplemented with 10% (v/v) 2-mercaptoethanol (Sigma-Aldrich, South Africa), and heated at 95 °C for 5 min. After loading the protein samples and the Page Ruler prestained protein ladder (5 µL) (Thermo Fisher Scientific, South Africa), the SDS-PAGE gel was run at 100 V for 95 min on the Mini-Protean Tetra Cell system (Bio-Rad, USA). The protein bands were visualized by staining the gel with Aqua Staining Solution (Vacutec, South Africa). Densitometry measurements were carried out using ImageJ v1.52a software (https://imagej.nih.gov/ij/download.html), which compares the optical densities of the target band of interest, against positive control bands [bovine serum albumin (BSA)] on the same gel. Twofold serial dilutions of BSA (Thermo Fisher Scientific, South Africa) were used for the generation of a standard curve of optical color intensity against the quantity of protein (µg), allowing for the yield of the SNAP-tag fusion protein to be estimated from the total protein concentration. Subsequently, western blotting was used to confirm the functionality and integrity of the recombinant his-tagged fusion protein. Protein bands were transferred from an unstained SDS-PAGE gel to a nitrocellulose membrane (PVDF transfer membrane, Roche, Switzerland) using a Mini Trans-Blot Cell system (Bio-Rad, USA) set at 100 V for 75 min. The membrane was then blocked with non-fat milk for 1 h at room temperature and incubated with a 1:1000 dilution of an anti-his rabbit primary antibody (Qiagen, Hilden, Germany) and a 1:5000 dilution of a goat anti-rabbit horseradish peroxidase (HRP)-conjugate antibody (Bio-Rad, USA). Clarity™ Western ECL substrate (Bio-Rad, USA) was added to the blot, prior to visualization using a Gel Doc™ XR Gel Documentation System (Bio-Rad, USA). A chemiluminescent ladder (5 µL) (SuperSignal™ Molecular Weight Protein Ladder, Thermo Fisher Scientific, South Africa) was used to assess the size of the protein bands.

### Conjugation of αCSPG4(scFv)-SNAP fusion protein to BG-modified substrates

#### Conjugation to BG-modified Alexa Fluor 488

The purified SNAP-tag based fusion protein (5 µM) (in 1 × PBS as the buffer system) was mixed with 10 µM of SNAP-Surface^®^ Alexa Fluor^®^ 488 (BG-Alexa Fluor 488, New England Biolabs, USA), 1 mM dithiothreitol (DTT, a reducing agent that improves the stability of SNAP-tag) (Sigma-Aldrich, South Africa) and made up to a final volume of 50 µL with 1 × PBS. Conjugation was carried out at 37 °C in the dark for 60 min. The labeled proteins were resolved on an SDS-PAGE gel and visualization of the fluorescent signal was made possible upon exposure to blue light excitation, using a Dark Reader Transilluminator (Clare Chemical Research, USA). The coupling efficiency was photometrically determined (as outlined by Hussain et al. 2019) using the theoretical extinction coefficients of the proteins and the extinction coefficients of the fluorescent dyes.

#### Generation of AURIF-containing immunoconjugates

Monomethyl auristatin F (MMAF) was sourced from BrightGene Bio-Medical Technology (China) and the compound BG-modified to generate BG–linker–AURIF by Professor Roger Hunter’s organic synthesis group, Department of Chemistry, University of Cape Town, South Africa. Detailed information about the synthesis, spectroscopic, and analytical data for the BG–linker–AURIF product is provided in Huysamen et al. (2023) (supplementary information). The purified recombinant fusion protein was incubated for 4 h at room temperature, with a threefold molar excess of BG–linker–AURIF [initially in lyophilized form, BG–linker–AURIF was solubilized in 100% (v/v) dimethyl sulfoxide (DMSO) (Sigma-Aldrich, South Africa), 1 M ectoine (a protein-stabilizing compatible solute (Lippert and Galinski 1992) (Sigma-Aldrich, South Africa) in 1 × PBS and 1 mM DTT]. The unconjugated BG–linker–AURIF was removed using 10 K-sized Amicon filters (Sigma-Aldrich, South Africa) according to the manufacturer’s instructions. Since the resulting product cannot be directly visualized, saturation of the binding domain of SNAP-tag with BG–linker–AURIF was ascertained by post-incubation (double conjugation) with a twofold molar excess of BG-Alexa Fluor 488 for 1 h at 37 °C. Next, SDS-PAGE analysis was conducted, and visualization of any potential fluorescence signal was carried out as described previously.

### Binding analysis and internalization of the αCSPG4(scFv)-SNAP-tag-based fusion proteins

#### Screening of target cells/FFPE tissue sections and validation of surface binding and internalization by confocal microscopy

Target cell lines (1 × 10^4^ cells) were seeded on a coverslip in a 35 mm dish and incubated in RPMI-1640 (Gibco #10566) or DMEM medium (Gibco #61870) (both supplemented with 10% (v/v) heat-inactivated FBS and 1% (v/v) 100 U/mL penicillin–streptomycin) overnight in a 5% CO_2_ incubator with 95% humidity at 37 °C. The next day, cells were incubated for 15–20 min with 15 µM of Alexa488-conjugated fusion protein in 100 µL of serum-free medium and 200 µL of 1:5000 Hoechst stain (Thermo Fisher Scientific, South Africa). Excess dye was removed by washing the cells three times with 1 × PBS, before fixing with 4% (v/v) paraformaldehyde (PFA) (Sigma-Aldrich, South Africa) for 20 min at room temperature. The cells were then washed one more time with 1 × PBS and the coverslip was mounted on a microscope slide (using Mowiol mounting medium from Merck, USA). The slides were left to dry in the dark at room temperature for 24 h, before images were captured on the Zeiss confocal-scanner microscope (LSM880) with Airyscan (Confocal and Light Microscope Imaging Facility, University of Cape Town, South Africa) on the 40X air objective.

Moreover, live cell imaging was conducted on tumor cell lines to assess the internalization and lysosomal localization of the αCSPG4(scFv)-SNAP fusion protein. For these experiments, 2 × 10^4^ Hs578T (CSPG4-positive) and 4 × 10^4^ MCF-7 (CSPG4-negative breast carcinoma) cells were seeded in each quadrant of 35 mm dishes. The tumor cells were incubated overnight at 37 °C and 95% humidity with 5% CO_2_ in supplemented media. The fusion protein was conjugated to BG-Alexa 488 as described in “Conjugation to BG-modified Alexa Fluor 488”. Here, 200 µL conjugation reactions, consisting of 5 µM αCSPG4(scFv)-SNAP, were prepared for each cell line and allowed to conjugate at 37 °C under dark conditions for 60 min. The conjugated αCSPG4(scFv)-SNAP-Alexa488 was then sterilized using 0.22-micron syringe filters and mixed thoroughly with 200 µL unsupplemented medium under sterile conditions. Once the cells were ready for staining, the media was removed, and the cells were washed with sterile 1 × PBS. The αCSPG4(scFv)-SNAP-Alexa488 in media was added to the respective quadrants (400 µL per quadrant) and allowed to incubate for 30 min at 37 °C. Next, unsupplemented media (400 µL per quadrant) was added to the designated unstained controls. Thereafter, 50 ng of LysoTracker (Molecular Probes; Thermo Fisher Scientific) was added and incubated at 37 °C for 30 min. Finally, the nuclear stain, Hoechst (diluted 1:5000 in media), was added and allowed to incubate at room temperature for 10 min. The cells were washed thrice with 1 × PBS in between all incubation steps, and supplemented media was added before visualization on the Zeiss LSM 880 confocal microscope (37 °C, 5% CO_2_) with a 63 × oil objective. All images were captured for further analysis using the Zeiss ZEN lite software (v3.6).

In parallel, 5 tissue sections were taken from 5 historical triple-negative patient samples. Each section was 5 mm thick and was cut from each patient block for immunofluorescence staining. Slides were placed in a vertical rack and dewaxed overnight at 60 °C in an incubator to carry out the labeling experiment. The sections from formalin-fixed paraffin-embedded (FFPE) tissues were deparaffinized and rehydrated prior to antigen retrieval performed by boiling in T-EG buffer (10 mM Tris, 0.5 mM EGTA, pH 9.0). Thereafter, 5 µM of the fusion protein was mixed with 10 µM of SNAP-Surface^®^ Alexa Fluor^®^ 647 (BG-Alexa Fluor 647, New England Biolabs, USA), 1 mM DTT and made up to a final volume of 50 µL with 1 × PBS. Here, the use of BG-Alexa647 was preferred as opposed to BG-Alexa488; BG-Alexa647 is a bright near-infrared (NIR) fluorescent dye which permits the exclusion of background staining on tissue sections. Conjugation was carried out at 37 °C in the dark for 60 min. This cocktail mixture was then used to label the tissue sections, which were incubated at 37 °C under a 5% CO_2_ atmosphere for 30 min and counterstained with Hoechst 33342 as per the NEB protocol. The confocal microscope (LSM 510 Zeiss, Confocal and Light Microscope Imaging Facility, University of Cape Town, South Africa) was used to scan tissue sections and Zen 2009 software was used to identify αCSPG4(scFv)-SNAP binding using area profiling. These settings were kept consistent throughout the data acquisition step. Confocal lasers scanned each pixel of the specimen gathering data about the αCSPG4(scFv)-SNAP intensity fluorescence. These fluorescent signal intensities were recorded for 5 manually identified tumor and non-tumor regions per section per patient and pooled for statistical analysis. Normalization of the data was carried out using the mean fluorescent intensity of an autofluorescent control (only DAPI stain used) for each patient sample. Statistical analysis was performed using GraphPad Prism 5. Data were expressed as mean ± standard deviation (SD). Statistical comparisons were made using a one-way ANOVA (****p < 0.0001).

#### Binding analysis and flow cytometric determination of receptor density on tumor cells

- Staining of cells

Briefly, adherent cells were lifted using Accutase^®^ solution (Sigma-Aldrich, South Africa). Approximately 2 × 10^6^ cells were washed twice in 2 mL of 1 × PBS to ensure complete removal of unspecific proteins in solution. The cell pellet was resuspended in 50 µL of a 1:2000 dilution of LIVE/DEAD™ amine-reactive, fixable Violet Dead Cell Stain Kit (ViViD) (product number: L34963, Thermo Fisher Scientific, South Africa) and incubated at room temperature for 20 min in the dark. The cells were then washed twice with 2 mL of FACS buffer [2% (v/v) FBS and 0.1% (v/v) sodium azide in 1 × PBS]. The cells were incubated with Alexa Fluor 488-conjugated protein in a total volume of 50 µL for 30 min at room temperature and washed twice with FACS buffer as previously described. Here, an antibody titration was carried out (in duplicate), with serial dilutions of αCSPG4(scFv)-SNAP-Alexa488 (0, 1, 5, 10, 25, 50, 100, 250, 500, and 1000 µg/mL). Subsequently, the cells were fixed with 1% (v/v) PFA solution and incubated for 10 min at room temperature in the dark. The cells were washed twice with 2 mL of 1 × PBS, resuspended in 300 µL of 1 × PBS, and kept in the dark at 4 °C until acquisition.

- Compensation controls

A total of 3 compensation tubes were used in this study: (1) unstained anti-mouse Ig-*Kappa* compensation beads (Becton–Dickinson (BD) Biosciences, USA) (negative compensation control), (2) anti-mouse Ig-*Kappa* compensation beads stained with 5 µL of 1:10 dilution of fluorescein isothiocyanate (FITC) mouse anti-human CD107 (BD Biosciences, USA) (single stain FITC/Alexa488 compensation control), and (3) ArC™ amine-reactive compensation beads (Thermo Fisher Scientific, South Africa) stained with 1 µL of 1:40 dilution of ViViD (Pacific Blue/ViViD compensation control). One drop of beads was stained with the appropriate antibody or dye and incubated for 30 min in the dark at room temperature. The beads were washed with 2 mL of 1 × PBS and resuspended in 150 µL of 1 × PBS and stored at 4 °C in the dark until acquisition.

- Acquisition

Samples were acquired using FACSDiva™ software (v8.0.1) (BD Biosciences, USA) on a BD™ LSR II flow cytometer (provided by the IDM Flow Cytometry Core Facility, University of Cape Town, South Africa). For each cell sample, 5 × 10^4^ events were acquired. Additionally, 2 × 10^4^ events were acquired for each compensation control.

- Analysis

Data analysis was carried out using FlowJo™ software (v10.6.1) (BD Biosciences, USA) and involved the generation of pseudocolor plots, whereby an appropriate gating strategy was devised to allow determination of the level of receptor expression on the surface of target cells (Fig. 4a). This also allowed the generation of antibody titration curves (using GraphPad Prism v5) for each cell line and αCSPG4(scFv)–SNAP–Alexa488 combinations, depicting the change in frequency and median fluorescence intensity (MFI) of the Alexa488-positive population. After selection of the optimal antibody titer, histograms and bar graph were generated, to compare the distribution of CSPG4 across target cell lines. Statistical analyses were performed using GraphPad Prism v5; Student’s *t* tests (relative to the negative cell line) were calculated to show any statistical difference. A *p* value of < 0.05 was considered to be statistically significant.

### Cytotoxicity studies

Cells (5 × 10^3^) were seeded in a 96-well plate (in either RPMI-1640 (Gibco #10566) or DMEM medium (Gibco #61870), supplemented with 10% (v/v) heat-inactivated FBS and 1% (v/v) 100 U/mL penicillin–streptomycin) and allowed to adhere overnight under standard tissue culture conditions (37 °C, 5% CO_2_ and 95% humidity). The next day, they were treated with threefold serially diluted concentrations of MMAF (unmodified), BG–linker–AURIF, αCSPG4(scFv)-SNAP (unconjugated) or αCSPG4(scFv)-SNAP-AURIF and incubated for 72 h in a 5% CO_2_ incubator with 95% humidity at 37 °C. Untreated cells served as negative controls (100% cell viability), while Zeocin-treated (100 µg/mL) cells were used as positive controls (0% cell viability). The Cell Proliferation Kit II (XTT) (product number: 11465015001, Roche, Switzerland) was used (according to the manufacturer’s protocol) to assess cytotoxicity. In this assay, cleavage of the tetrazolium salt XTT occurs in the presence of metabolically active cells and results in the formation of orange formazan crystals, which absorb light at 450 nm. Thus, at 68 h post-treatment, cells were treated with the XTT reagent and at 72 h, absorbance readings (at 450 nm as the measurement filter and 650 nm as the reference filter) were taken using a spectrophotometer (iMark™ Microplate Absorbance Reader, Bio-Rad, USA). All experiments were carried out in triplicate (*n* = 3), with 3 technical repeats. The absorbance values were normalized with respect to the untreated and Zeocin controls, and the results were presented as a percentage of cell viability. The concentration required to achieve a 50% reduction in cell viability (IC_50_ value) was calculated using GraphPad Prism v5 software.

## Results

### Engineering and expression of αCSPG4(scFv)-SNAP

**Fig. 1:**
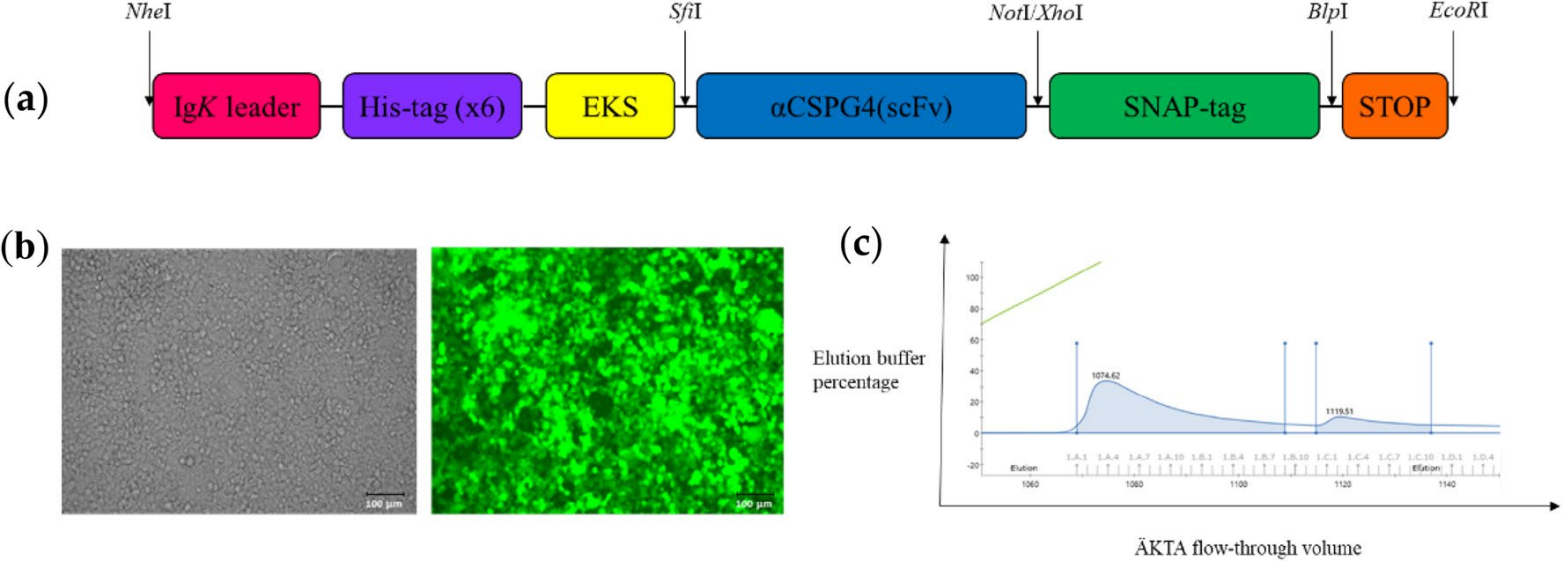
Generation of αCSPG4(scFv)-SNAP. **a** ORF coding for αCSPG4(scFv)-SNAP. Here, unique *Sfi*I and *Not*I restriction sites were used in the cloning of the scFv genes into the pCB-SNAP mammalian expression vector. Important components of the ORFs include: Ig*K* (Ig-*Kappa*) leader sequence for secretion of the fusion protein expressed by host cells; His-tag (×6), 6 histidine tags for protein purification by IMAC and detection in western blot analysis; EKS (enterokinase cleavage site) for the enzymatic removal of the N-terminal elements and STOP, a stop codon for halting protein synthesis); **b** microscopic visualization of eGFP in HEK293T cells transfected with pCB-αCSPG4(scFv)-SNAP DNA. Enrichment was performed using 100 µg/mL of Zeocin. The green channel (right panel) was used to assess eGFP expression, while the brightfield (or phase contrast) channel (left panel) showed the number of cells in a specific region. Images were taken using a ZOE™ Fluorescent Cell Imager at 100 µm magnification; **c** chromatogram of αCSPG4(scFv)-SNAP after purification using IMAC. The *y*-axis is a measure of the elution buffer percentage, while the *x*-axis represents the ÄKTA flow-through volume with respect to increasing time. The blue line shows the elution profile of fusion protein, and the green line demonstrates the concentration gradient of imidazole. Fractions were eluted in the form of two distinct peaks on the chromatogram

The variable heavy chain (*V*_H_) and variable light chain (*V*_L_) gene sequences from the complementarity-determining regions (CDRs) of the CSPG4-specific mAb9.2.27 were arranged in a scFv format. In silico cloning allowed genetic modification of the scFv through insertion of unique restriction enzyme cutting sites. The open-reading frame (ORF) for the construct was accordingly generated through incorporation of the scFv sequence into the eGFP-expressing pCB-SNAP mammalian expression vector, which includes various components that are essential to generating the final active product (Fig. 1a). Following molecular cloning, the selected recombinant plasmid clones were verified by the conventional Sanger sequencing before proceeding to protein expression using HEK293T cells (Fig. 1b). Since the transfection efficiency was less than 70–80%, Zeocin selection (100 µg/mL) was applied to enrich the eGFP-positive cell population carrying the bleomycin resistance gene. These cells were then maintained in culture (ideally at 90% confluency) during collection of the cell culture supernatant (containing the fusion protein of interest).

Purification of the recombinant fusion protein (from 1 L of cell culture supernatant) was carried out using Immobilized Metal Affinity Chromatography (IMAC), whereby the fusion protein was eluted through competitive binding between his-tag and imidazole for the Ni^2+^ column. The resulting elution profile is shown in Fig. 1c, depicting the tendency of the purified SNAP-tag-based fusion protein being eluted from the column in the form of two distinct peaks, upon the application of increasing imidazole concentration via a gradient (0–30% imidazole) and step elution process (100% imidazole).

### Protein validation and characterization of IMAC-purified αCSPG4(scFv)-SNAP

**Fig. 2:**
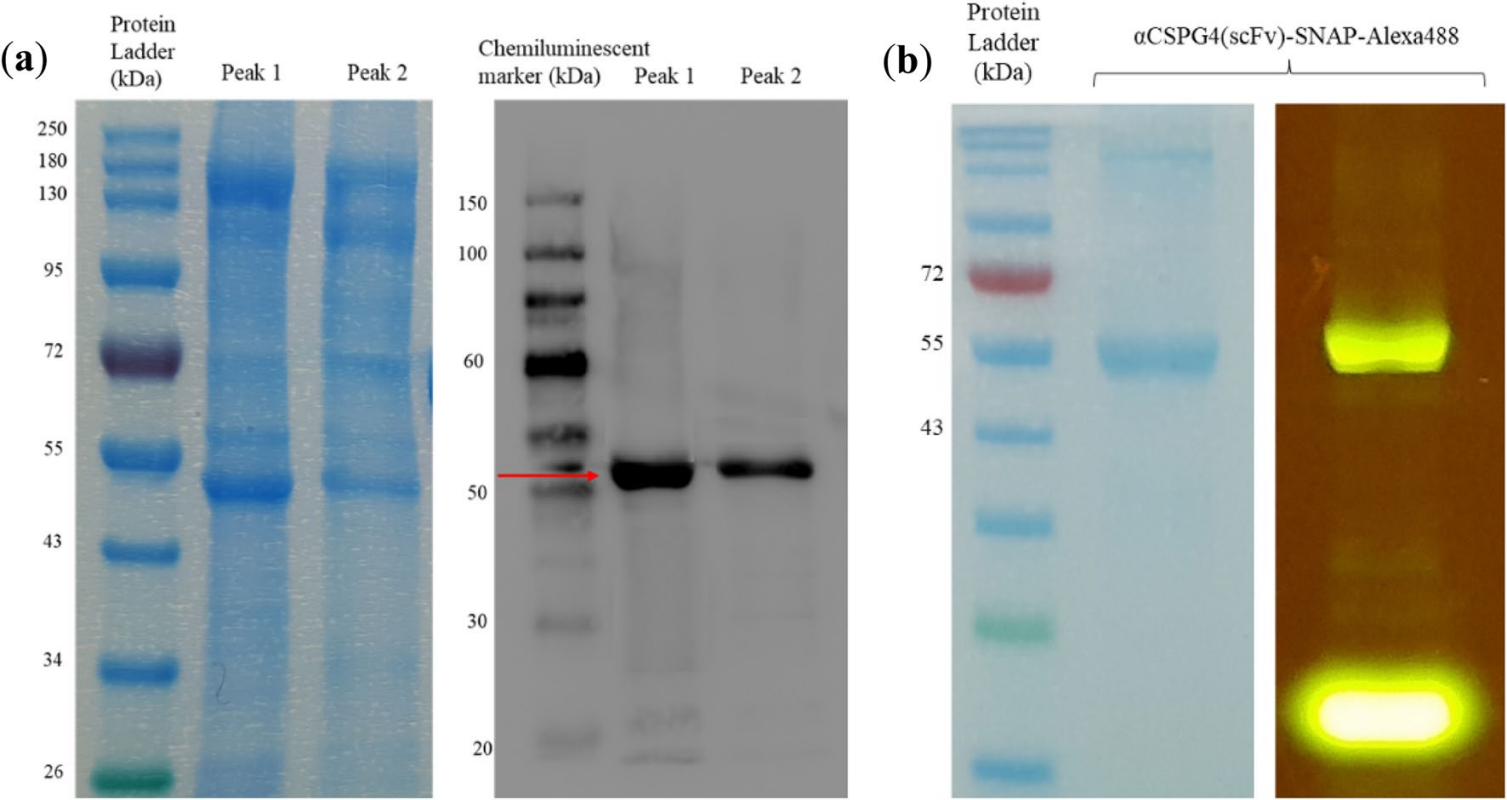
Characterization of IMAC-purified αCSPG4(scFv)-SNAP. **a** SDS-PAGE and western blot analysis of enriched αCSPG4(scFv)-SNAP (51.1 kDa) protein fractions. Left panel: comparison of protein profiles on a 10% SDS-PAGE gel stained with Aqua staining solution. Right panel: immunoblot of proteins transferred to a nitrocellulose membrane from a duplicate SDS-PAGE gel. A chemiluminescent ladder (SuperSignal™ Molecular Weight Protein Ladder) was used to assess the size of the protein bands. An anti-his rabbit antibody (1:1000 primary antibody) and a goat anti-rabbit HRP-conjugate antibody (1:5000 secondary antibody) were used. Red arrow indicates presence of full-length recombinant scFv-SNAP fusion protein. The membrane was visualized using a Gel Documentation System; **b** assessing the binding activity of αCSPG4(scFv)-SNAP to BG-Alexa Fluor 488. A ratio of 1:2 of protein to BG-Alexa Fluor 488 was used in the conjugation reaction. Left panel: Alexa488-conjugated protein ran on a 10% SDS-PAGE gel stained with Aqua staining solution. Right panel: the same SDS-PAGE gel visualized under blue light for potential fluorescence. A Dark Reader Transilluminator was used for visualization of the fluorescent signal

Following IMAC purification, the resulting, concentrated protein fractions (from peaks 1 and 2) were separated on a 10% SDS-PAGE gel [Fig. 2a (left panel)] and the fusion protein was identified based on its molecular weight [theoretical size for αCSPG4(scFv)-SNAP is 51.1 kDa]. The presence of additional non-specific bands necessitated densitometry measurements to estimate the protein concentration which corresponds to the scFv–SNAP fusion protein. To this end, a standard curve was plotted, consisting of the optical color intensity of the BSA standards (quantified by ImageJ software) on the *y*-axis, against the corresponding amount of protein on the *x*-axis (data not shown). By extrapolating the measured color intensity of the target bands to their protein quantity, the calculated yields of each protein peak were determined, indicating an absolute yield ranging from 0.75 to 2.35 mg/L, respectively. Such prevailing poor yield advocates the need for an additional or improved protein purification strategy. Nonetheless, since the lowest values were obtained from peak 2, peak 1 was rationally chosen for use in the downstream functional assays.

Successful expression of the full-length recombinant αCSPG4(scFv)-SNAP fusion protein-bearing functional his-tags was further confirmed by western blot analysis [Fig. 2a (right panel)]. The membrane was incubated with an anti-his rabbit primary antibody which binds to the C-terminal histidine residues of the fusion protein. A secondary antibody (goat anti-rabbit HRP-conjugate antibody) then allowed visualization of the his-tagged fusion protein by reaction with a chemiluminescent substrate. From Fig. 2a, presence of intact full-length fusion protein observed on the SDS-PAGE gel (left panel) can be correlated to the same molecular size bands on the western blot (as indicated by red arrow on right panel).

After confirming the N-terminal integrity of the recombinant fusion protein, the self-labeling activity of the SNAP-tag moiety was analyzed. The αCSPG4(scFv)-SNAP fusion protein was conjugated with BG-Alexa Fluor 488 as described in “Conjugation to BG-modified Alexa Fluor 488”, and visualized under blue light excitation on a 10% SDS-PAGE gel (Fig. 2b). Correspondence with the theoretical size of the fusion protein confirms the functionality and binding activity of SNAP-tag to BG-modified substrates such as BG-Alexa Fluor 488. The bottom band indicated the presence of unconjugated (excess) fluorescent substrate, which was removed by size exclusion chromatography before proceeding to binding assays. The coupling efficiency was determined as described previously (Hussain et al. 2019) and after 30 min incubation at 37 °C, the resulting labeling efficiency was 91% for αCSPG4(scFv)-SNAP.

### Investigating the binding and internalization of αCSPG4(scFv)-SNAP-Alexa Fluor 488/647 on target cells

#### Screening of target cells and validation of surface binding and internalization by confocal microscopy

**Fig. 3:**
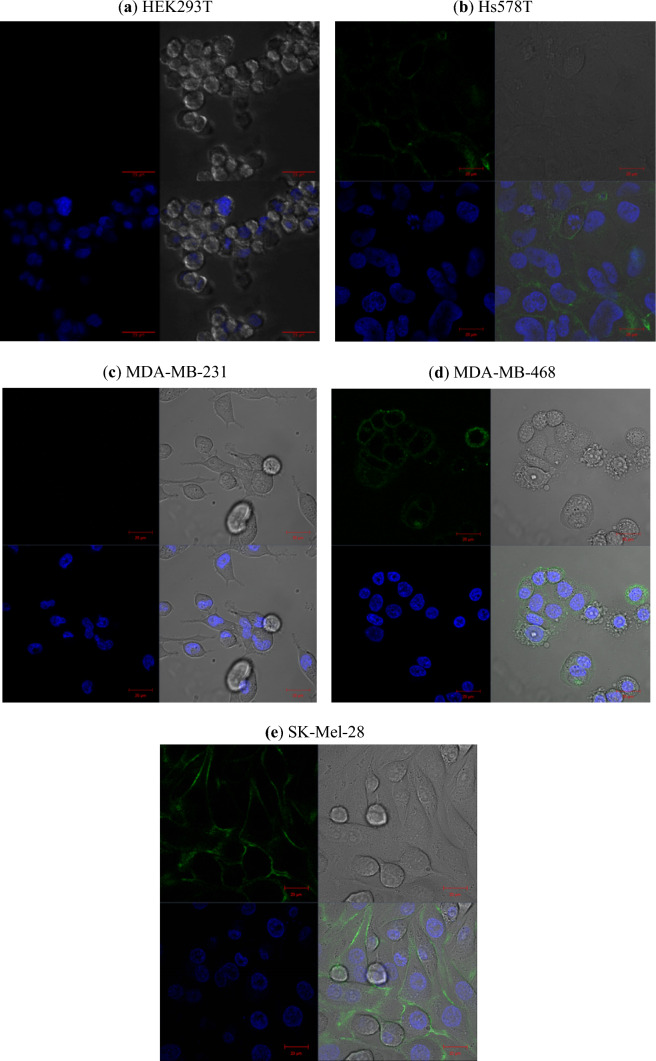
Assessing the binding activity of αCSPG4(scFv)-SNAP-Alexa488 by screening target cells for potential CSPG4 expression. **a** HEK293T, **b** Hs578T, **c** MDA-MB-231, **d** MDA-MB-468, and **e** SK-Mel-28. Cell lines were incubated with 15 µM of conjugated protein (green signal) for 15–20 min at 37 °C. Hoechst (1:5000 dilution in media) was used as a stain for the nuclei (blue signal). Washes were performed three times with 1 × PBS, before fixing with 4% PFA and mounting the coverslips on a microscope slide. Images were captured using a Zeiss confocal-scanner microscope (LSM880) with Airyscan at 20 µm magnification

The overarching working principle of targeted diagnostics and therapeutics relies heavily on the expression of accessible and often surface-bound target antigens. Consequently, to fully assess the potential of the SNAP-tag-based immunoconjugate as therapeutics for TNBCs, it became instrumental to assess the membrane expression of CSPG4 on a panel of target cell lines (consisting of Hs578T, MDA-MB-231, and MDA-MB-468 as TNBC cells) using confocal microscopy. Simultaneously, this also allowed the binding ability (or functional integrity) of the antibody fragment to be evaluated. Membrane binding of αCSPG4(scFv)-SNAP-Alexa488 (Fig. 3) (green signal) was confirmed on Hs578T, MDA-MB-468, and SK-Mel-28 cells (melanoma cells).

**Fig. 4:**
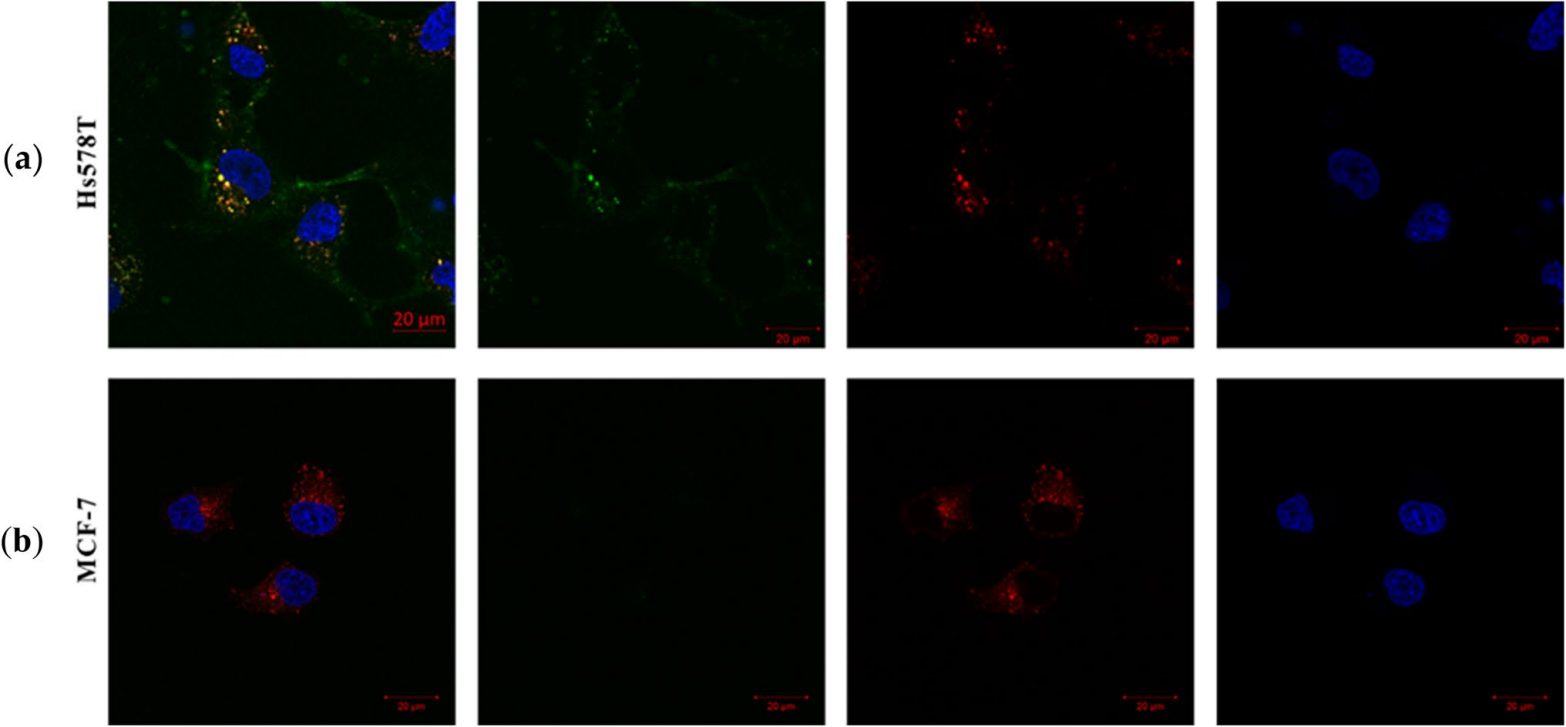
Live-cell imaging to assess internalization and lysosomal routing of the αCSPG4(scFv)-SNAP fusion protein conjugated to Alexa 488. **a** Antigen-positive Hs578T and **b** antigen-negative MCF-7 tumor cell lines were incubated with 5 µM αCSPG4(scFv)-SNAP-Alexa488 (green signal) for 30 min at 37 °C. The lysosomal compartments were stained with 50 ng of LysoTracker (red signal) for 30 min at 37 °C. The nuclei were counterstained with Hoechst (blue signal) diluted 1:5000 in media. Images were captured with the Zeiss confocal-scanner microscope (LSM880) with Airyscan at ×63 magnification

Interestingly, Hs578T and SK-Mel-28 cells displayed even membrane staining throughout most fields of view, MDA-MB-468 cells showed staining in certain areas only, and HEK293T and MDA-MB-231 cells exhibited no CSPG4-associated fluorescent signal. Therefore, the latter two cell lines were postulated to be negative for CSPG4. Additionally, specific internalization of αCSPG4(scFv)-SNAP-Alexa488 by the antigen-positive Hs578T cells was detected using confocal microscopy. After the initial 30 min incubation at 37 °C with αCSPG4(scFv)-SNAP-Alexa488 and an additional 30-min incubation period at 37 °C with the LysoTracker, the signal of the fusion protein was detected at the cell surfaces as well as within the cells (Fig. 4). The distinct colocalization between the internalized fusion protein signal and the stained lysosomal signal indicates that after internalization, the αCSPG4(scFv)-SNAP-Alexa488 was localized within the lysosomal compartments of the cell.

**Fig. 5:**
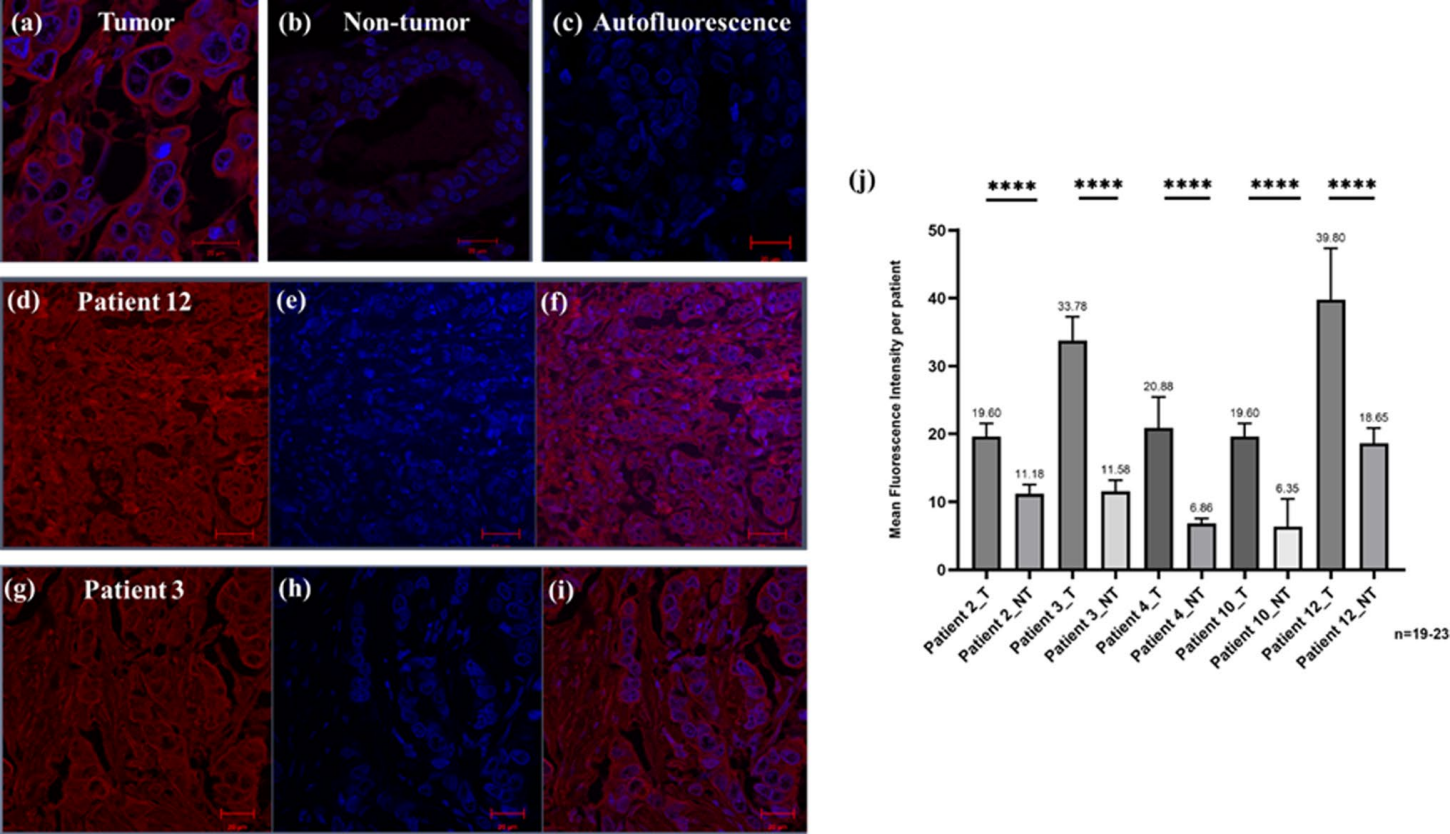
Binding of αCSPG4(scFv)-SNAP on South African breast cancer patient tissue sections and the pooled mean fluorescence intensities of CSPG4 expression. Using the LSM confocal 510 microscope, FFPE tissue sections were imaged. The mean of each patient’s fluorescence intensity data was extracted and tabulated for comparison. **a** αCSPG4(scFv)-SNAP conjugated to BG-Alexa647 which labels the cell membrane (in red) of a tumorigenic tissue section; **b** DAPI panel showing nuclear staining of cells (in blue) on a non-tumor sample and **c** auto-fluorescence control. The αCSPG4(scFv)-SNAP pooled label data for patient means were compared using one-way ANOVA (****p < 0.0001). The mean intensity data indicated significant differences between all tumor (T) and non-tumor (NT) tissues in the selected patient samples as shown in **j**. Qualitative differences are indicated as a comparison of the fluorescence image panels (as previously indicated) of patient 12 (**d**–**f**) and patient 3 (**g**–**i**). These samples were normalized against an autofluorescent control for each patient

Moreover, the binding potential of the antibody component of αCSPG4(scFv)-SNAP was further validated by staining FFPE breast cancer tissue sections of 5 South African patients (diagnosed with TNBC) and deriving the pooled mean fluorescence intensities of CSPG4 expression from the images generated and comparing these values against non-tumor tissue sections from the same patient (Fig. 5). Indeed, the immunofluorescence intensity results showed dense membrane binding of αCSPG4(scFv)-SNAP-Alexa647 in TNBC patient tissue sections as compared to the auto-fluorescence control and non-tumor tissues (Fig. 5a–c). Furthermore, CSPG4’s expression level in tumor tissue sections was significantly higher than that in non-tumor control tissue sections (Fig. 5j). There is a significant difference in CSPG4 expression between tumor (T) versus non-tumor (NT) (*p* < 0.0001) breast tissues, with varying differences in CSPG4 expression between patient tumor tissue sections.

#### Binding analysis and flow cytometric determination of receptor density on tumor cells

**Fig. 6:**
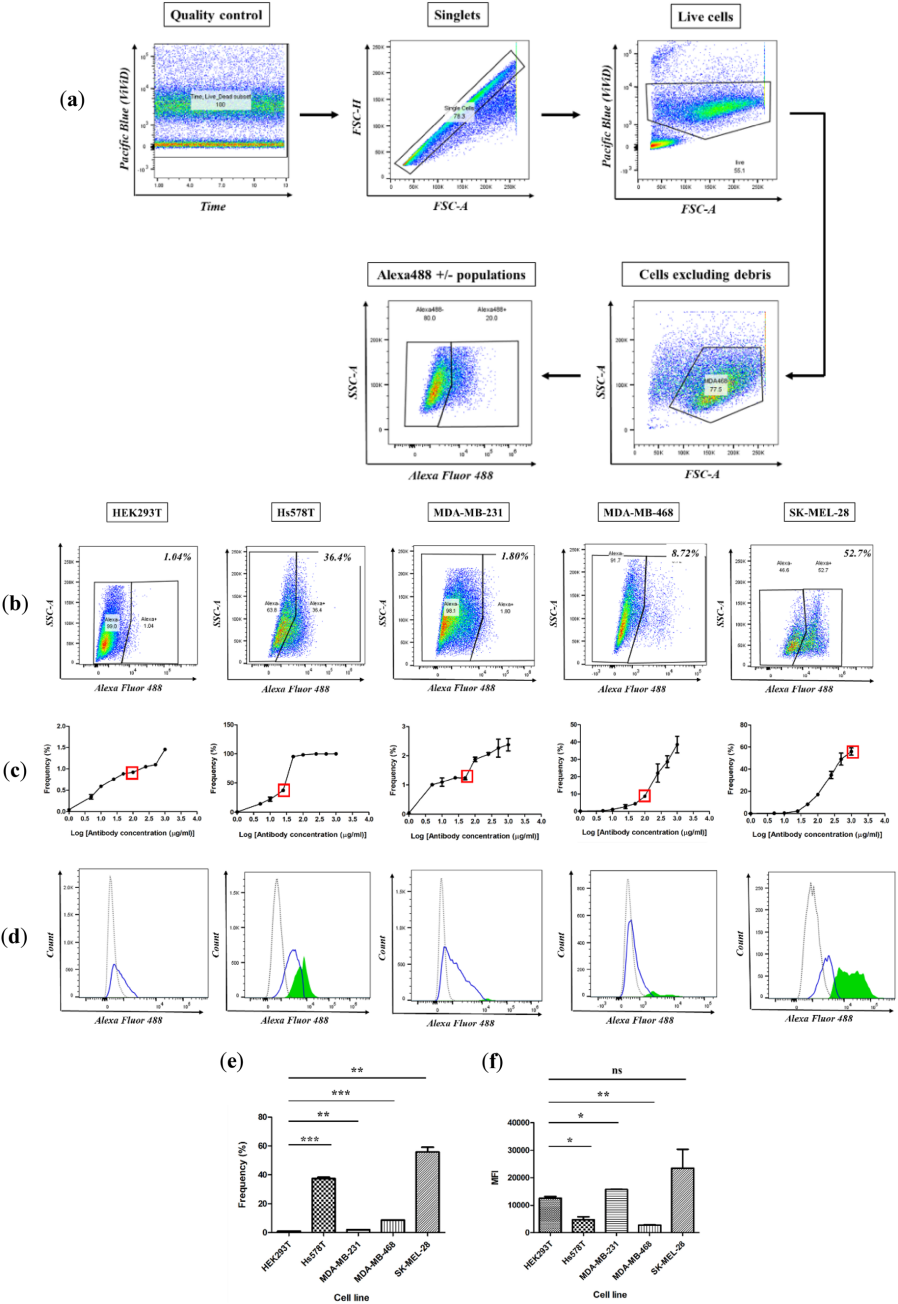
Comparison of the CSPG4 expression status across target cell lines. Cells were incubated with the optimal Alexa488-conjugated αCSPG4(scFv)-SNAP concentration and acquired on a BD™ LSR II flow cytometer. Data shown are representative of two biological repeat experiments. **a** Representative pseudocolor plots indicating the gating strategy employed in the determination of the receptor expression status; **b** representative pseudocolor plots with gates showing the position of the Alexa488-positive/negative cell populations at the optimal antibody titer. Frequencies of the Alexa488-positive populations (expressed as a percentage of the total population) are indicated at the top right-hand corner of the plots; **c** antibody titration curves showing the frequencies of the Alexa488-positive population at the optimal antibody concentration (indicated by red box); **d** histograms depicting the relative fluorescence of the Alexa488-positive/negative populations (gray curve: untreated cells, blue curve: Alexa488-negative cells at the optimal antibody concentration, green curve: Alexa488-positive cells at the optimal antibody concentration); bar graphs demonstrating the **e** frequencies of the Alexa488-positive population and **f** median fluorescence intensity (MFI). Statistical comparisons (relative to the CSPG4-negative HEK293T cell line) were calculated using Student’s *t* tests [*p < 0.05, **p < 0.01, ***p < 0.001, ns (not significant)]

Following confocal microscopy, flow cytometric analysis was implemented to further confirm the binding specificity and capability of the αCSPG4(scFv)-SNAP fusion protein, while synchronously quantifying surface expression of its cognate antigen within the live cell population. On this basis, the same panel of target cell lines used in “Screening of target cells and validation of surface binding and internalization by confocal microscopy” was subjected to incubation with various serial dilutions of the Alexa488-conjugated fusion protein, prior to flow cytometric analysis. Here, compensation (Roederer 2001; Szalóki and Goda 2015) was applied to correct for spectral overlap arising from the use of BG-Alexa Fluor 488 and ViViD (live/dead marker), which were detectable in both the Alexa488 and Pacific Blue channels. Thereafter, determination of the receptor expression status for each sample required the design and implementation of an appropriate gating strategy (Fig. 6a). As an essential optimization step (Hulspas 2010), antibody titrations were performed to estimate the optimal concentration of scFv-SNAP-Alexa488 needed to ensure the best segregation between the antigen-positive and negative populations for a given cell type. This strategy allowed determination of the antibody staining concentration to use to ensure the most accurate measure of expression levels, while limiting background interference.

Thereafter, quantitative analyses were performed to (1) evaluate the percentage of antigen-positive cells present within the cell population, (2) quantify the receptor distribution per cell type [via the median fluorescent intensity (MFI)], and (3) compare these values across the chosen panel of target cells (which includes TNBC cells) to confirm their suitability in cytotoxicity studies. As such, the percentages of CSPG4-expressing cells were 1.04, 36.4, 1.80, 8.72, and 52.7% for HEK293T, Hs578T, MDA-MB-231, MDA-MB-468, and SK-Mel-28 cells, respectively (Fig. 6b, c).

Additionally, histograms depicting the relative fluorescence shift suggest that, among the TNBC cell lines, Hs578T exhibited the highest count of CSPG4-associated signal, whereas MDA-MB-468 and MDA-MB-231 cells were characterized by medium-to-low signals, respectively (Fig. 6d). Reflecting the confocal microscopy observations, the frequency of the antigen-positive cells within the cell population was as follows: SK-Mel-28 > Hs578T > MDA-MB-468 > MDA-MB-231 > HEK293T. These results validated the concordance between the qualitative and quantitative binding data generated in this study (Table 1). Interestingly, statistical comparisons across cell lines revealed that the population of CSPG4-positive cells was significantly lower in HEK293T cells (Fig. 6e), making it an important negative control cell line for further functional assays.

**Table 1:** Overview of binding activities for Alexa Fluor 488-conjugated αCSPG4(scFv)-SNAP fusion protein on target cell lines

Cell lines tested	Surface binding as observed via confocal microscopy	Frequency of antigen-positive cells as determined by flow cytometry
HEK293T	No binding	1.04%
Hs578T	Uniform and intense	36.4%
MDA-MB-231	No binding	1.80%
MDA-MB-468	Mild and sparse	8.72%
SK-Mel-28	Uniform and intense	52.7%

Similarly, MFI values were calculated to establish the levels of antigen expression within the Alexa488-positive population (Fig. 6f). This is achievable, since the intensity of the fluorescent signal is proportional to the amount of antibody bound per cell, which is in turn reflective of the number of antigen sites expressed (Mittag and Tárnok 2009; Mizrahi et al. 2018). Surprisingly, CSPG4-positive Hs578T cells showed a low distribution of receptors on their surface despite exhibiting a frequency of 36.4% in the overall cell population. Unfortunately, the underlying causality behind these confounding observations is currently unknown and will require further investigation. Therefore, given its correlation with the confocal microscopy data, the computed frequency values were thus regarded as the best estimate of relative receptor abundance for each cell population.

### Cytotoxic analysis of SNAP-tag based fusion proteins conjugated to BG–linker–AURIF

After confirming the functionality of the individual elements of the αCSPG4(scFv)-SNAP fusion protein and determining the relative abundance of the antigen-positive cells across target cell lines, the next step involved establishing whether the fusion protein can be used as a vehicle for the specific delivery of cytotoxic agents to TNBC cells. Initially, with BG–linker–AURIF being synthesized from commercially available MMAF (BrightGene Bio-Medical Technology, China), an important requisite was to assess whether such chemical modifications had any impact on the cytotoxic activity of the resulting compound. To this end, all cell lines were treated with increasing concentrations of the drug and cell proliferation was measured using an XTT-based cell viability assay. The concentration of drug required to achieve 50% inhibition in cell viability was also calculated to assess drug efficacy. Indeed, Fig. S1 and S2 (supplementary information) demonstrate that both (unmodified) MMAF and BG–linker–AURIF were indiscriminately toxic toward all cell lines in a concentration-dependent fashion. This unspecific activity provided the rationale for using an antibody moiety as a guiding head for the targeted delivery of such warheads to tumor cells. Most importantly, MMAF displayed a more potent killing effect (IC_50_ range 43.0–453 nM) as compared to BG–linker–AURIF (IC_50_ range 32.0–17,100 nM) (Table 2). Consequently, despite this marginal reduction in activity (probably due to the smaller molecular size of MMAF which allows easier internalization into target cells), their overlapping range suggested that BG–linker–AURIF retained most of its anti-mitotic properties and was therefore suitable for use in the generation of novel recombinant ADCs targeting CSPG4.

**Table 2:** Summary of IC_50_ values based on the cytocidal activity of αCSPG4(scFv)-SNAP-AURIF, unmodified MMAF, and unconjugated BG–linker–AURIF on target cell lines

Treatment	Cell lines tested	IC_50_ value (nM)
αCSPG4(scFv)-SNAP-AURIF	HEK293T	–
	Hs578T	173.3
	MDA-MB-231	–
	MDA-MB-468	190.3
	SK-Mel-28	1660
BG–linker–AURIF	HEK293T	32.3
	Hs578T	846.1
	MDA-MB-231	81.6
	MDA-MB-468	181.9
	SK-Mel-28	17,100
Unmodified MMAF	HEK293T	57.6
	Hs578T	203.7
	MDA-MB-231	45.1
	MDA-MB-468	43.0
	SK-Mel-28	166.4

**Fig. 7:**
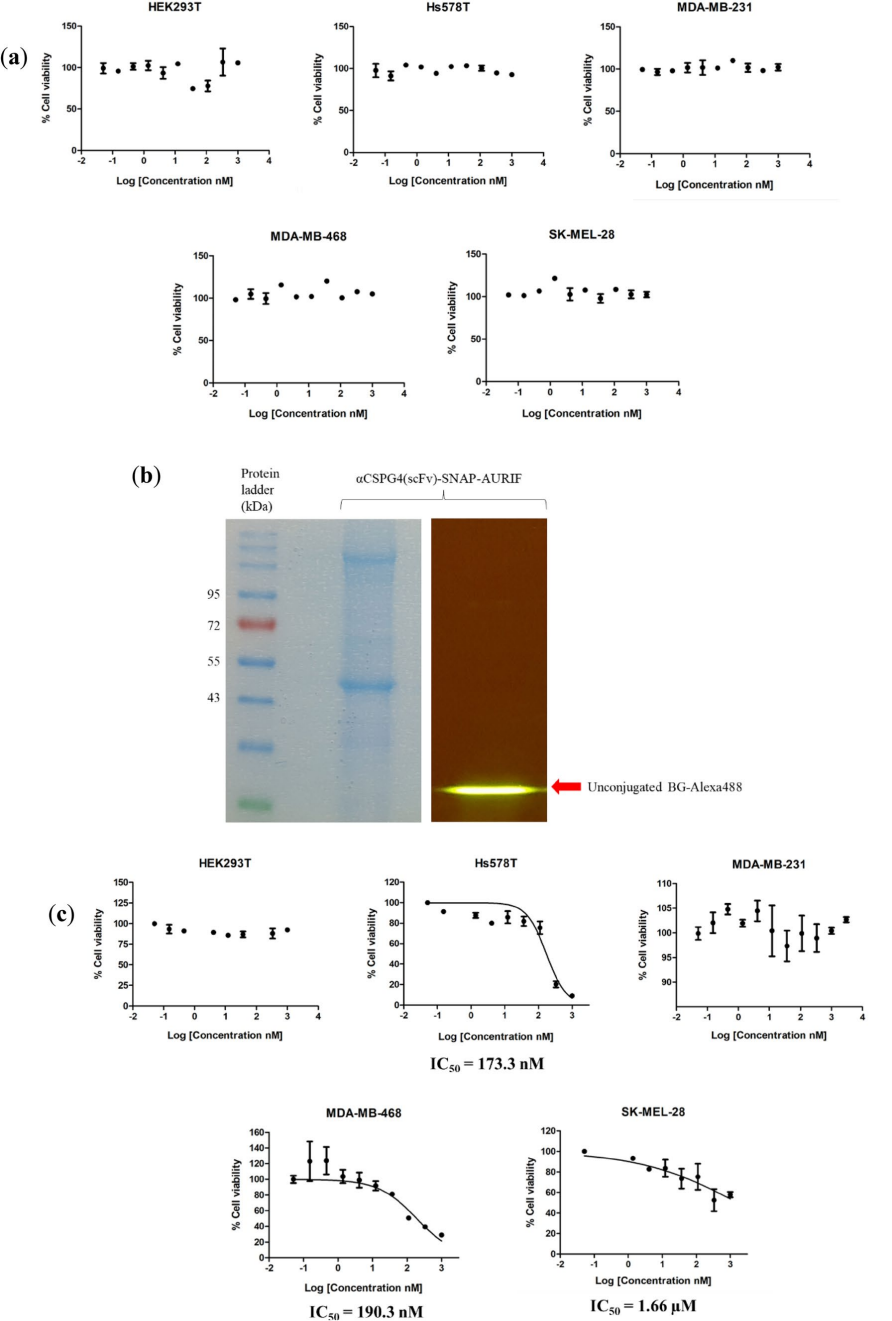
Evaluation of the cytotoxicity of AURIF-conjugated αCSPG4(scFv)-SNAP fusion proteins. **a** Unconjugated αCSPG4(scFv)-SNAP fusion protein displays negligible toxicity on target cell lines; **b** confirming the saturation of scFv-SNAP fusion proteins with BG–linker–AURIF through a double conjugation with BG-Alexa Fluor 488. After conjugation with BG–linker–AURIF for 4 h at room temperature, 5 µM of the conjugation reaction was incubated with 10 µM of BG-Alexa Fluor 488 for 60 min at 37 °C, before being loaded on a 10% SDS-PAGE gel, which was visualized under blue light using a Dark Reader Transilluminator (right panel) and stained using Aqua staining solution (left panel). Unbound BG-Alexa Fluor 488 is indicated by red arrow; **c** dose–response curves demonstrating the cytotoxic activity of αCSPG4(scFv)-SNAP-AURIF in vitro. The cytotoxic activity was assessed using an XTT-based viability assay after incubation with the drug for 72 h. Cells were treated with (threefold serially diluted) increasing concentrations of the drug and the IC_50_ values (relative to the untreated and zeocin-treated cells) were calculated using GraphPad Prism v5. Data are mean ± standard deviation (SD) of each measurement (presented as a percentage of cell viability), and the measurements were performed in triplicate at least three times

Subsequently, prior to conjugation with BG–linker–AURIF, it was necessary to first ascertain that the unconjugated fusion proteins were not responsible for any visible lethal effect on the target cells. As expected, treatment with unconjugated αCSPG4(scFv)-SNAP fusion protein did not induce any palpable cellular toxicity (Fig. 7a). Following conjugation to BG–linker–AURIF, a second conjugation was performed with the addition of BG-Alexa Fluor 488, to confirm saturation of the scFv-SNAP with BG–linker–AURIF prior to cytotoxicity studies. As shown in Fig. 7b, no fluorescent signal was detected (right panel), despite the presence of the conjugated protein on the stained SDS-PAGE gel (left panel). On this account, incubation of the fusion protein with a threefold molar excess of BG–linker–AURIF for 4 h at room temperature was deemed to be sufficient to allow complete saturation of the binding domain of SNAP-tag with the antiproliferative drug.

After 72 h incubation with incremental concentrations of αCSPG4(scFv)-SNAP-AURIF, cells that presented with a high frequency of CSPG4-expressing cells (Hs578T, MDA-MB-468, and SK-Mel-28) were reduced in a concentration-dependent manner (Fig. 7c). The concentration required to achieve a 50% reduction in TNBC cell viability (IC_50_ value) was as follows: 173.3 nM for Hs578T and 190.3 nM for MDA-MB-468 (Table 2). However, in the case of the melanoma cell line SK-Mel-28, higher drug concentrations were required to generate a dose–response curve that would allow a reliable calculation of the IC_50_ value. Nonetheless, with the observable low protein yield (“Protein validation and characterization of IMAC-purified αCSPG4(scFv)-SNAP”), extrapolation of the graph was favored (using GraphPad Prism v5), which allowed the determination of an estimated IC_50_ value of 1.66 µM for SK-Mel-28. In contrast, cell lines that were less abundant in antigen-positive cells (HEK293T and MDA-MB-231 cells) were negligibly affected, thereby demonstrating that the resulting AURIF cytotoxicity is dependent on the level of CSPG4 expression. Moreover, any potential cytotoxic effects induced by the unspecific toxicity of AURIF were factored out using an isotype control (αASPH(scFv)-SNAP-AURIF) on CSPG4^+^ Hs578T cells (Fig. S3). These data further confirmed that the in vitro cytotoxicity of the recombinant ADC is dependent on the specificity for the target antigen. Interestingly, although SK-Mel-28 cells exhibited the highest frequency (52.7%) of CSPG4-positive cells (Fig. 6e), they were less sensitive to the treatment as opposed to Hs578T and MDA-MB-468 cells. This unanticipated observation suggested that the biological activity and efficiency of such compounds were dictated by additional in vitro considerations. Taken together, the resulting IC_50_ values generated from the AURIF-driven cytotoxicity studies in this section are summarized in Table 2.

## Discussion

CSPG4 has been recognized as a potential target in cancer immunotherapy, as it is minimally expressed in healthy tissues, while being overexpressed in several cancers, including TNBC, the most aggressive breast cancer subtype majoritarily affecting patients of African descent (Hu et al. 2022; Hoffmann et al. 2020; Eng et al. 2018; Amoury et al. 2016a, b). TNBC tumors do not inherently express ER, PgR, and HER2 receptors and are therefore limited to different chemotherapy treatment regimens to which they rapidly develop resistance, causing an observed limited median overall survival (OS) of approximately 14.5 months, in comparison to their HER2-enriched (50.1 months) and luminal (42.9 months) breast cancer-bearing patient counterparts (Hu et al. 2022). Consequently, it becomes evident that alternative therapeutic treatments are needed. Targeted immunotherapy using ADC, exploiting the specificity of mAbs and the differential expression of TAAs, to dispatch chemically conjugated cytotoxic compounds within targeted cells, represents a viable therapeutic option, having demonstrated clinical benefits in treating HER2-expressing breast cancer patients, as illustrated by the FDA approval of many ADCs, including trastuzumab emtansine (Kadcyla, Genentech, 2013) and trastuzumab deruxtecan (Enhertu, Daiichi Sankyo/AstraZeneca, 2019). However, to achieve their therapeutic fruitions, most ADCs rely on the expression of clinically relevant cancer biomarkers such as CSPG4, whose co-expression with the well characterized programmed death-ligand-1 (PDL-1: immune checkpoint inhibitor) in 25% of TNBC patients bearing TP53 mutation, has been associated with poor prognosis, treatment response, and overall survival, respectively (Hu et al. 2022).

This study set to develop a αCSPG4(scFv)-SNAP-tag based antibody fusion protein to specifically detect and kill CSPG4-expressing TNBC cells. This fusion protein was successfully generated through the genetic fusion of the CSPG4-specific mAb9.2.27 scFv fragments with SNAP-tag, enabling the development of a reproducible and novel conjugation method with predictable stoichiometry. SNAP-tag is a self-labeling engineered mutant of the human O(6)-alkylguanine-DNA alkyltransferase enzyme, capable of specifically and covalently reacting with any BG-substrates, in an irreversible manner to generate homogeneous conjugates in a 1:1 stoichiometric reaction (Amoury et al. 2016a, b; Biteghe et al. 2020a, b; Woitok et al. 2017; Holliger and Hudson 2005; Asaadi et al. 2021). This technology offers several advantages to traditional conjugation methods that rely on lysine side chains or reduced disulfides residues to arm mAbs with cytotoxic payloads, thus generating heterogeneous products with varied DAR and pharmacokinetics behaviors, which can potentially lead to serious life-threatening side effects in patients (Hussain et al. 2019; Hamblett et al. 2004; Junutula et al. 2008; Shefet-Carasso and Benhar 2015; Beerli et al. 2015; Beckley et al. 2013; Adem et al. 2014). Furthermore, the selection of the scFv over their mAb counterparts stems from the facts that they have shown to possess several advantages including improved pharmacokinetic features related to their enhanced ability to efficiently penetrate blood vessels walls and solid tumors; abrogate unwanted side effects due to Fc portion deprivation and rapid clearance from the bloodstream in virtue of their short half-life (0.5–2 h) related to their size (Muñoz-López et al. 2022). Of late, scFvs account for close to 35% of all antibody fragments currently used in clinical trials (NCT00272181, NCT00412776 and NCT02449239).

The SNAP-tag based fusion proteins can be generated through various production system ranging from yeast, bacteria, and mammalian systems such as HEK293T cells as previously reported (Amoury et al. 2016a, b; Kampmeier et al. 2009; Hussain et al. 2011; Woitok et al. 2016; von Felbert et al. 2016). In this regard, a αCSPG4(scFv)-SNAP fusion protein was collected and purified from tissue culture supernatant with a yield of approximately 2.35 mg/L. Furthermore, the low transfection efficiencies (< 70–80%) observed from the lipid-based transfection method employed suggest that this method can be substituted by alternative, well-established methods (Thomas and Smart 2005; Nimesh and Chandra 2008). Despite these suboptimal conditions, sufficient amounts of CSPG4-targeting SNAP-tag-based fusion protein were generated and enriched, capable of efficiently self-labeling with a twofold molar excess of BG-Alexa 488 and selectively binding to high (Hs578T and SK-Mel-28 cells) and moderate (MDA-MB-468 cells) CSPG4-expressing cells, as well as tumorigenic TNBC FFPE tissue sections. These results corroborate the previous reports demonstrating the specific binding of both αCSPG4(scFv)-SNAP-IR700 antibody-photoconjugate (antibody attached to a light sensitive NIR dye) and the αCSPG4(scFv)-MAP human cytolytic fusion protein (antibody fused to a human cytolytic enzyme) to CSPG4-expressing TNBC cells. However, surface binding was observed on MDA-MB-231 and Hs578T cells, but not to MDA-MB-468 cells, respectively (Amoury et al. 2016a, b). This observed dichotomy in CSPG4 expression levels between the MDA-MB-231 and MDA-MB-468 in this study and previous reports could be related to various physiological conditions, which potentially modulate receptor expression. For instance, CSPG4 has been found to be expressed within the very dynamic cancer stem cell (CSC) population representing 1–5% of tumors, in many cancers (Yang et al. 2004; Cooney et al. 2011; Wang et al. 2010a, b). These CSCs are highly tumorigenic, slow-growing therapeutic resistant cells, mostly contributing to tumor heterogeneity through their self-renewal capacity and ability to recapitulate the parental phenotype of treated tumors, causing most clinically observed tumor recurrence or relapses (Yang et al. 2009; Cooney et al. 2011; Wang et al. 2010a, b; Touil et al. 2013). Hence, these CSC attributes could partially explain the discrepancy in CSPG4 expression status in these different cell lines, as this phenotype is strongly associated with different tumor growth phases and poor prognosis (Hu et al. 2022). Moreover, it can be speculated that several environmental and physiological factors play a defining role in the modulation of receptor dynamics within tumor cells. Undoubtedly, while immortal cell lines constitute a cost-effective and effortless method to study biological processes, they also represent very artificial systems (lacking close cell-to-cell contact), which may not adequately mimic primary tumor cells. In the same line, long-term serial passages of cell lines can induce genotypic and phenotypic variations in the cell population (Kaur and Dufour 2012), which can rise to further heterogeneity and inconsistencies in the data set. Similarly, different cell detachment methods have been reported to cause a discrepancy in cell-surface receptor expression between experiments or studies (Lai et al. 2022; Nowak-Terpiłowska et al. 2021) For example, trypsinization, a method characterized by the proteolytic activity of trypsin enzyme, is mostly used for cellular dissociation and detachment. However, this cell detachment method has been found to significantly degrade cell-surface proteins and the extracellular matrix (Lai et al. 2022; Nowak-Terpiłowskaet al. 2021). Taking this into consideration, accutase was used throughout this study in virtue of its limited proteolytic activity to a small number of cell-surface proteins, such as CD163 and CD206 (Lai et al. 2022; Nowak-Terpiłowska et al. 2021). Yet, accutase was recently reported to temporary suppress both FasL and Fas receptors, which could bias the interpretation of the cell death-related experimental results, given the prominent role they play in apoptosis induction (Lai et al. 2022). Therefore, it is of paramount importance to select the appropriate cell detachment method, which best fit the study targets and experimental design, since the effect of accutase on FasL is reversible, as highlighted by the recovery of FasL normal cell-surface expression 24 h post-treatment (Lai et al. 2022).

On the other hand, it is noteworthy to mention that the key to successful management of breast cancer revolves around early screening and detection (Ping et al. 2012; Brooks et al. 2009). Based on the aggressive nature of TNBCs, accurate diagnosis becomes vital for determining prognosis and ensuring delivery of the optimal therapy for patients (Penault-Llorca and Viale 2012). Traditionally, TNBC diagnosis is dictated by immunohistochemistry (IHC) (to assess ER, PgR, and HER2 status), although in clinical practice, it is often a two-step process, combining morphological imaging and IHC to identify specific cancer biomarkers, which can help predict potential treatment outcomes or provide the best clinically available targeted therapy (Wang et al. 2008; Gao et al. 2014; Wang et al. 2021; Sanchez et al. 2021). To address this critical aspect of cancer diagnosis (and precision medicine), the fluorescent-IHC capacity of the αCSPG4(scFv)-SNAP fusion protein was demonstrated by its ability to specifically bind the CSPG4-expressing cells on FFPE tumor biopsies of South African TNBC patients, thereby distinguishing healthy from TNBC tissues based on superior expression levels of CSPG4 in the latter. These findings were important and correlated with our previous reports confirming the photoimmunodiagnostic potential of αCSPG4(scFv)-SNAP-Alexa647 in binding specifically to FFPE TNBC biopsies of Caucasian patients (Amoury et al. 2016a). As such, these SNAP-tag-based fusion proteins can potentiate the concomitant detection and quantification of several target proteins (biomarkers) on TNBC biopsies and those of other cancers, and subsequently offer a therapeutic avenue, when substituting their diagnostic imaging agent with a therapeutic cytotoxic molecule as previously described (Amoury et al. 2016a, b; Biteghe et al. 2020a, b; Woitok et al. 2017; von Felbert et al. 2016). Additionally, this study provides further insight into the mechanism of action of the αCSPG4(scFv)-SNAP fusion protein, which is specifically trafficked to the sub-cellular lysosomal compartment following its internalization via receptor-mediated endocytosis. It is important to note that ADCs generally rely on lysosomal degradation to dissociate the payload from the antibody through proteolytic cleavage and to subsequently release it into the cytosol to exert its cytotoxic effect (Chalouni and Doll 2018; Firer and Gellerman 2012). This mechanism is reflective of AURIF-based ADCs, whereby AURIF is released following proteolytic lysosomal degradation to access the microtubules in the cytosol to induce apoptosis (Gauzy-Lazo et al. 2020).

Based on these premises, this study aimed to evaluate the targeted cytotoxic profile of αCSPG4(scFv)-SNAP fusion protein, on CSPG4-expressing TNBC cells post-site-specific conjugation with a single AURIF derivative, thereby generating novel recombinant (next-generation) ADCs. Monomethyl auristatin E (MMAE) is an anti-mitotic cytotoxic drug, structurally derived from Dolastatin-10, a novel pentapeptide agent and is the most commonly used cytotoxic payload in ADCs, while MMAF is a more hydrophobic derivative of the compound and possesses a charged C-terminal phenylalanine residue, which facilitates reduced membrane permeability, thus attenuating bystander effects (cytotoxic effects on adjacent cells) observed with its MMAE counterpart (Bouchard et al. 2014; Kim and Kim 2015; H. Li et al. 2016a, b; Sommer et al. 2016; Rizzo et al. 2022; Theunissen et al. 2018; Smith et al. 2006). These agents act by halting tubulin polymerization, causing the disruption of microtubule assembly by limiting the kinetics and length of the polymers, thus activating cell cycle arrest (G2/M arrest), culminating in the induction of apoptotic cell death (Bai et al. 1990a, b; Kim and Kim 2015; Bouchard et al. 2014). BG modification of MMAF has been shown to effectively induce apoptosis at nanomolar concentrations in breast cancer and human solid tumor cells when conjugated to recombinant scFv fragments targeting HER2 and EGFR (Woitok et al. 2016, 2017; Huysamen et al. 2023). Furthermore, imaging studies confirm that treatment with the MMAF ADCs leads to microtubule disassembly in conjunction with decreased cell viability (Woitok et al. 2017; Best et al. 2021).

In concordance with the previous reports, our study revealed the capacity of our αCSPG4(scFv)-SNAP fusion protein to efficiently couple with BG–linker–AURIF to generate αCSPG4(scFv)-SNAP-AURIF. Consequently, the selective cytotoxic potential of αCSPG4(scFv)-SNAP-AURIF was confirmed by exposing target cells with increasing concentrations of our immunoconjugate which selectively reduced cell viability in a dose-dependent manner, with IC_50_ values ranging from 173.3 to 1660 nM. Of note, these IC_50_ concentrations negligibly affected minimally expressing CSPG4 MDA-MB-231 and the HEK293T control cells, thereby highlighting the specific intracellular delivery of AURIF within target cells. As demonstrated by the previous studies, scFv-SNAP fusion proteins are unable to induce cell killing on their own (Fig. 7a) (Biteghe et al. 2020b; Woitok et al. 2016; von Felbert et al. 2016), implying that the observed cytotoxic effect is achieved through the anti-mitotic activities of the AURIF component attached to the fusion protein. The results obtained further refine existing evidence that conjugation of diverse functional groups (such as fluorophores, photosensitizers, and toxins) to SNAP-tag does not compromise the binding specificity of the antibody, nor prevent its internalization (Biteghe et al. 2020b; Woitok et al. 2016; von Felbert et al. 2016).

Interestingly enough, unconjugated BG–linker–AURIF gave rise to IC_50_ values ranging between 32.3 nM and 17.1 µM, thus confirming that BG modification of the toxin does not impair its cytotoxicity (Woitok et al. 2016). In agreement with several findings (Woitok et al. 2017, 2016; Aubrey et al. 2018), these IC_50_ values (treatment with BG–linker–AURIF alone) are higher than those observed with the conjugated fusion protein, validating the fact that targeted drug delivery increases potency and efficacy. As compared to state-of-the-art BG-AURIF commercialized by Tube Pharmaceuticals (Vienna, Austria) (Woitok et al. 2017)) which relies on the direct attachment of AURIF to BG, a novel aspect of this study pertains to the use of a BG-PEG_3_-N_3_ piece to couple AURIF onto SNAP-tag (Huysamen et al. 2023). The novel click chemistry involved allows distancing of the AURIF molecule from the active domain (or binding pocket) of SNAP-tag, while generating a more hydrophilic immunoconjugate product with enhanced water solubility and improved conjugation efficiency. Furthermore, contrary to the IC_50_ values obtained in this study, higher cytotoxic activities (0.6–12 nM) were obtained with previous scFv-SNAP-AURIF recombinant ADCs (Woitok et al. 2017, 2016). A possible explanation for this discrepancy might relate to the difference in expression levels of the target receptors. Likewise, the purity of the fusion proteins might come into play; the lower the amount of competing degradation product in the recovered protein sample, the lower the expected IC_50_ values. Meanwhile, the flow cytometric analysis conducted in this study, may not be the most accurate reflection of the spatial and temporal receptor dynamics exhibited by tumor cells used in the toxicity studies. Research thus far on the correlation between cell-surface receptor expression and activity of ADCs remains contradictory, suggesting the involvement of multiple mechanisms in modulating ADC activity (Koga et al. 2015; Sommer et al. 2016; Li et al. 2013; Polson et al. 2010)). Indeed, it was found that the intracellular concentration of the released payload was linked with in vitro ADC-mediated cytotoxicity, independent of target expression (Li et al. 2016a, b). This also means that patient selection relying solely on antigen expression may not guarantee ADC anti-tumor efficacy and, therefore, additional tumor markers or immune-derived relevant biomarkers may be required to select patients for ADC therapy (Tang et al. 2019).

Multiple resistance mechanisms can also affect the potency and pharmacology of ADCs; for instance, downregulation of cell-surface antigens reduces antibody binding, while elevated levels of drug transporters can limit the effectiveness of the payload (Loganzo et al. 2016; Goler-Baron and Assaraf 2012; Imai et al. 2012). Due to these multiple variable parameters, it becomes increasingly difficult to compare the activity of different ADCs across cell lines (Li et al. 2016a, b). ADCs as potential targeted delivery systems (for solid tumors) must be able to overcome all hurdles (Nejadmoghaddam et al. 2019), including traveling through the bloodstream and penetrating the tumor mass. Of note, an unstable linkage can result in premature release of the toxic payload prior to reaching the diseased site (Khandelwal et al. 2013). Hence, achieving reasonable chemical stability is instrumental when designing the linkage between the antibody and the effector component. In congruity with the previous reports (Woitok et al. 2017, 2016), we showcase the application of SNAP-tag technology as a method to achieve stable and efficient linkage of the small molecule toxin AURIF to scFvs targeting CSPG4 surface antigen. Since the in vitro cytotoxic effects of ADCs correlate with the number of effector molecules on the antibody (Pillow et al. 2014), multiple AURIF derivatives could be attached to one BG molecule and conjugated to SNAP-tag to potentially improve the efficacy of the recombinant ADCs, without affecting their antigen-binding ability or their homogeneity (Woitok et al. 2017, 2016). Alternatively, the introduction of a cleavable linker in-between the scFv and SNAP-tag (such as the cathepsin B cleavable citrulline–valine linker in brentuximab vedotin) could also enhance cytotoxicity by assisting in the intracellular release of the toxic payload (Woitok et al. 2016). This PEG-linker addition is commonly used as a stabilizer to reduce scFv clearance rate from bloodstream, thus increasing their half-life and potency (Muñoz-López et al. 2022). In this regards, 425(scFv)-SNAP-tag conjugated with near-infrared dye (BG-747) was reported to specifically accumulate in the tumor 10 h post-injection, with a rapid complete bloodstream clearance observed 72 h post-injection (Kampmeier et al. 2010).

To further reduce the potential adverse events associated with our AURIF conjugate, αCSPG4(scFv)-SNAP-tag based photoimmunotherapy (PIT) could be prioritized for non-metastatic triple-negative breast tumors on the basis of PIT requiring an extra step of light activation to exert its phototoxic effects (Amoury et al. 2016a; Hsu et al. 2022). Consequently, AURIF-based SNAP-tag conjugates will potentially only be offered for metastatic TNBCs, having disseminated to secondary organs or tissues, which are inaccessible to therapeutic light (Biteghe et al. 2020b; Hsu et al. 2022; Jin et al. 2016). Overall, this work represents a first important step in the establishment of methodologies geared toward the engineering of more effective ADCs, paving the way to the next generation of ADCs addressing clinically relevant hurdles faced in precision medicine.

## Conclusion

Overall, this study showcases the rapid and preliminary analysis of SNAP-tag-based fusion proteins which provides the foundation for the development of a catalogue of companion immunodiagnostic tools and their corresponding therapeutics, thereby providing a much-needed incentive for a shift toward personalized medicine in the management of TNBC patients. For instance, ex vivo binding of SNAP-tag-based fusion proteins to breast cancer biopsies could enable the identification and quantification of relevant triple-negative breast tumor markers (through multiplex immunofluorescence imaging), to recognize and classify patients according to their ability to benefit from a specific treatment plan. Due to the absence of stand-alone drugs in the battle against cancer, this process will simultaneously expedite the implementation of a multi-pronged approach involving the synergistic effect of the best treatment options aimed toward providing a curative benefit.

## Supplementary Information

Below is the link to the electronic supplementary material.

Supplementary file1 (DOCX 526 KB)

## Data Availability

Not applicable.

## References

[CR1] Adem YT, Schwarz KA, Duenas E (2014). Auristatin antibody drug conjugate physical instability and the role of drug payload. Bioconjug Chem.

[CR2] Amoury M, Bauerschlag D, Zeppernick F (2016). Photoimmunotheranostic agents for triple-negative breast cancer diagnosis and therapy that can be activated on demand. Oncotarget.

[CR3] Amoury M, Mladenov R, Nachreiner T (2016). A novel approach for targeted elimination of CSPG4-positive triple-negative breast cancer cells using a MAP tau-based fusion protein. Int J Cancer.

[CR4] Asaadi Y, Jouneghani FF, Janani S, Rahbarizadeh F (2021). A comprehensive comparison between camelid nanobodies and single chain variable fragments. Biomark Res.

[CR5] Aubrey N, Allard-vannier E, Martin C (2018). Site-specific conjugation of auristatins onto engineered scFv using second generation maleimide to target HER2-positive breast cancer in vitro ´. Bioconjug Chem.

[CR6] Bai R, Pettit GR, Hamel E (1990). Structure-activity studies with chiral isomers and with segments of the antimitotic marine peptide dolastatin 10. Biochem Pharmacol.

[CR7] Bai R, Pettit GR, Hamel E (1990). Dolastatin 10, a powerful cytostatic peptide derived from a marine animal. Inhibition of tubulin polymerization mediated through the vinca alkaloid binding domain. Biochem Pharmacol.

[CR8] Beckley NS, Lazzareschi KP, Chih HW (2013). Investigation into temperature-induced aggregation of an antibody drug conjugate. Bioconjug Chem.

[CR9] Beerli RR, Hell T, Merkel AS, Grawunder U (2015). Sortase enzyme-mediated generation of site-specifically conjugated antibody drug conjugates with high In Vitro and In Vivo potency. PLoS ONE.

[CR10] Best RL, LaPointe NE, Azarenko O (2021). Microtubule and tubulin binding and regulation of microtubule dynamics by the antibody drug conjugate (ADC) payload, monomethyl auristatin E (MMAE): mechanistic insights into MMAE ADC peripheral neuropathy. Toxicol Appl Pharmacol.

[CR11] Biteghe FAN, Mungra N, Chalomie NET (2020). Advances in epidermal growth factor receptor specific immunotherapy: lessons to be learned from armed antibodies. Oncotarget.

[CR12] Biteghe FAN, Chalomie NET, Mungra N (2020). Antibody-based immunotherapy: alternative approaches for the treatment of metastatic melanoma. Biomedicines.

[CR13] Bouchard H, Viskov C, Garcia-Echeverria C (2014). Antibody−drug conjugates—a new wave of cancer drugs. Bioorg Med Chem Lett.

[CR14] Brooks AD, Nover AB, Jagtap S (2009). Modern breast cancer detection: a technological review. Int J Biomed Imaging.

[CR15] Chalouni C, Doll S (2018). Fate of antibody−drug conjugates in cancer cells. J Exp Clin Cancer Res.

[CR16] Chen DS, Mellman I (2017). Elements of cancer immunity and the cancer-immune set point. Nature.

[CR17] Coleman N, Yap TA, Heymach JV (2023). Antibody−drug conjugates in lung cancer: dawn of a new era?. NPJ Precis Oncol.

[CR18] Cooney CA, Jousheghany F, Yao-borengasser A et al (2011) Chondroitin sulfates play a major role in breast cancer metastasis : a role for CSPG4 and CHST11 gene expression in forming surface P-selectin ligands in aggressive breast cancer cells 10.1186/bcr2895 PMC3218947 21658254

[CR19] Dolan ME, Moschel RC, Pegg AE (1990). Depletion of mammalian O6-alkylguanine-DNA alkyltransferase activity by O6-benzylguanine provides a means to evaluate the role of this protein in protection against carcinogenic and therapeutic alkylating agents. Proc Natl Acad Sci USA.

[CR20] Eng MS, Kaur J, Prasmickaite L (2018). Enhanced targeting of triple-negative breast carcinoma and malignant melanoma by photochemical internalization of CSPG4-targeting immunotoxins. Photochem Photobiol Sci.

[CR21] Ferlay J, Colombet M, Soerjomataram I (2021). Cancer statistics for the year 2020: an overview. Int J Cancer.

[CR22] Firer MA, Gellerman G (2012). Targeted drug delivery for cancer therapy: the other side of antibodies. J Hematol Oncol.

[CR23] Fitting J, Blume T, Ten HA (2015). Phage display-based generation of novel internalizing antibody fragments for immunotoxin-based treatment of acute myeloid leukemia. Mabs.

[CR24] Ford CHJ, Newman CE, Johnson JR (1983). Localisation and toxicity study of a vindesine-anti-CEA conjugate in patients with advanced cancer. Br J Cancer.

[CR25] Gao B, Zhang H, Zhang SD (2014). Mammographic and clinicopathological features of triple-negative breast cancer. Br J Radiol.

[CR26] Gauzy-Lazo L, Sassoon I, Brun MP (2020). Advances in antibody−drug conjugate design: current clinical landscape and future innovations. SLAS Discov.

[CR27] Ghosh A, Syed SM, Kumar M (2020). In vivo cell fate tracing provides no evidence for mesenchymal to epithelial transition in adult fallopian tube and uterus. Cell Rep.

[CR28] Goler-Baron V, Assaraf YG (2012). Overcoming multidrug resistance via photodestruction of ABCG2-rich extracellular vesicles sequestering photosensitive chemotherapeutics. PLoS ONE.

[CR29] Hamblett KJ, Senter PD, Chace DF (2004). Effects of drug loading on the antitumor activity of a monoclonal antibody drug conjugate. Clin Cancer Res.

[CR30] Harris JM, Chess RB (2003). Effect of pegylation on pharmaceuticals. Nat Rev Drug Discov.

[CR31] Hoffmann RM, Crescioli S, Mele S (2020). A novel antibody−drug conjugate (ADC) delivering a DNA mono-alkylating payload to chondroitin sulfate proteoglycan (CSPG4)-expressing melanoma ricarda. Cancers (Basel).

[CR32] Holliger P, Hudson PJ (2005). Engineered antibody fragments and the rise of single domains. Nat Biotechnol.

[CR33] Hsu MA, Okamura SM, De MCD (2022). Cancer-targeted photoimmunotherapy induces antitumor immunity and can be augmented by anti-PD-1 therapy for durable anticancer responses in an immunologically active murine tumor model. Cancer Immunol Immunother.

[CR34] Hu Z, Fan C, Oh DS (2006). The molecular portraits of breast tumors are conserved across microarray platforms. BMC Genomics.

[CR35] Hu Z, Zheng C, Yang J (2022). Co-expression and combined prognostic value of CSPG4 and negative breast cancer. Front Oncol.

[CR36] Hueper WC (2021). An insight into FDA approved antibody−drug conjugates for cancer therapy. Molecules.

[CR37] Hulspas R (2010). Titration of fluorochrome-conjugated antibodies for labeling cell surface markers on live cells. Curr Protoc Cytom.

[CR38] Hussain AF, Kampmeier F, Von Felbert V (2011). SNAP-tag technology mediates site specific conjugation of antibody fragments with a photosensitizer and improves target specific phototoxicity in tumor cells. Bioconjug Chem.

[CR39] Hussain AF, Krüger HR, Kampmeier F (2013). Targeted delivery of dendritic polyglycerol-doxorubicin conjugates by scFv-SNAP fusion protein suppresses EGFR+ cancer cell growth. Biomacromol.

[CR40] Hussain AF, Heppenstall PA, Kampmeier F (2019). One-step site-specific antibody fragment auto-conjugation using SNAP-tag technology. Nat Protoc.

[CR41] Huysamen AM, Fadeyi OE, Mayuni G (2023). Click chemistry-generated auristatin F–linker–benzylguanine for a SNAP-tag-based recombinant antibody−drug conjugate demonstrating selective cytotoxicity toward EGFR-overexpressing tumor cells. Am Chem Soc Omega Omega.

[CR42] Imai Y, Yamagishi H, Ono Y, Ueda Y (2012). Versatile inhibitory effects of the flavonoid-derived PI3K/Akt inhibitor, LY294002, on ATP-binding cassette transporters that characterize stem cells. Clin Transl Med.

[CR43] Jin J, Krishnamachary B, Mironchik Y (2016). Phototheranostics of CD44-positive cell populations in triple negative breast cancer. Sci Rep.

[CR44] Junutula JR, Raab H, Clark S (2008). Site-specific conjugation of a cytotoxic drug to an antibody improves the therapeutic index. Nat Biotechnol.

[CR45] Kampmeier F, Ribbert M, Nachreiner T, Dembski S, Beaufils F, Brecht ABS (2009). Site specific, covalent labeling of recombinant antibody frag ments via fusion to an engineeredversion of 6-O-alkylguanine DNA alkyltransferase. Bioconjug Chem.

[CR46] Kampmeier F, Niesen J, Koers A (2010). Rapid optical imaging of EGF receptor expression with a single-chain antibody SNAP-tag fusion protein. Eur J Nucl Med Mol Imaging.

[CR47] Kaur G, Dufour JM (2012). Cell lines valuable tools or useless artifacts. Spermatogenesis.

[CR48] Keppler A, Kindermann M, Gendreizig S (2004). Labeling of fusion proteins of O6-alkylguanine-DNA alkyltransferase with small molecules in vivo and in vitro. Methods.

[CR49] Khandelwal A, Saber H, Shapiro MZH (2013). Antibody−drug conjugates and immunotoxins: from pre-clinical development to therapeutic applications. New York Hum Press.

[CR50] Kim EG, Kim KM (2015). Strategies and advancement in antibody−drug conjugate optimization for targeted cancer therapeutics. Biomol Ther.

[CR51] Koga Y, Manabe S, Aihara Y (2015). Antitumor effect of antitissue factor antibody-MMAE conjugate in human pancreatic tumor xenografts. Int J Cancer.

[CR52] Korde LA, Somerfield MR, Carey LA (2021). Neoadjuvant chemotherapy, endocrine therapy, and targeted therapy for breast cancer: ASCO guideline. J Clin Oncol.

[CR53] Lai TY, Cao J, Ou-Yang P (2022). Different methods of detaching adherent cells and their effects on the cell surface expression of Fas receptor and Fas ligand. Sci Rep.

[CR54] Li D, Poon KA, Yu S (2013). DCDT2980S, an anti-CD22-monomethyl auristatin E antibody–drug conjugate, is a potential treatment for non-hodgkin lymphoma. Mol Cancer Ther.

[CR55] Li F, Emmerton KK, Jonas M (2016). Intracellular released payload influences potency and bystander-killing effects of antibody−drug conjugates in preclinical models. Cancer Res.

[CR56] Li H, Yu C, Jiang J (2016). An anti-HER2 antibody conjugated with monomethyl auristatin E is highly effective in HER2-positive human gastric cancer. Cancer Biol Ther.

[CR200] Lippert K, Galinski EA (1992) Enzyme stabilization be ectoine-type compatible solutes: protection against heating, freezing and drying. Appl Microbiol Biotechnol 37:61–65. 10.1007/BF00174204

[CR57] Loganzo F, Sung M, Gerber HP (2016). Mechanisms of resistance to antibody−drug conjugates. Mol Cancer Ther.

[CR58] McCombs JR, Owen SC (2015). Antibody drug conjugates: design and selection of linker, payload and conjugation chemistry. AAPS J.

[CR59] Mittag A, Tárnok A (2009). Basics of standardization and calibration in cytometry—a review. J Biophotonics.

[CR60] Mizrahi O, Ish Shalom E, Baniyash M, Klieger Y (2018). Quantitative flow cytometry: concerns and recommendations in clinic and research. Cytom Part B Clin Cytom.

[CR61] Muñoz-López P, Ribas-Aparicio RM, Becerra-Báez EI, Fraga-Pérez K, Flores-Martínez LF, Mateos-Chávez AA, Luria-Pérez R (2022). Single-chain fragment variable: recent progress in cancer diagnosis and therapy. Cancers.

[CR01] Nasiri H, Valedkarimi Z, Aghebati-Maleki L, Majidi J (2018) Antibody-drug conjugates: promising and efficient tools for targeted cancer therapy. J Cell Physiol 233(9):6441–6457. 10.1002/jcp.26435 10.1002/jcp.26435 PMC13482507 29319167

[CR63] Natali P, Bigotti A, Cavalieri R (1985). Distribution of a cross-species melanoma-associated antigen in normal and neoplastic human tissues. J Invest Dermatol.

[CR64] Nejadmoghaddam MR, Minai-Tehrani A, Ghahremanzadeh R (2019). Antibody−drug conjugates: possibilities and challenges. Avicenna J Med Biotechnol.

[CR65] Nimesh S, Chandra R (2008). Guanidinium-grafted polyethylenimine: an efficient transfecting agent for mammalian cells. Eur J Pharm Biopharm.

[CR66] Nowak-Terpiłowska A, Śledziński P, Zeyland J (2021). Impact of cell harvesting methods on detection of cell surface proteins and apoptotic markers. Braz J Med Biol Res.

[CR67] Pegg AE, Dolan ME, Moschel RC (1995). Structure, function, and inhibition of O6-alkylguanine-DNA alkyltransferase. Prog Nucleic Acid Res Mol Biol.

[CR68] Penault-Llorca F, Viale G (2012). Pathological and molecular diagnosis of triple-negative breast cancer: a clinical perspective. Ann Oncol.

[CR69] Pillow TH, Tien J, Parsons-Reponte KL (2014). Site-specific trastuzumab maytansinoid antibody−drug conjugates with improved therapeutic activity through linker and antibody engineering. J Med Chem.

[CR70] Ping Wu, Gao Y, Hui Zhang CC (2012). Aptamer-guided silver-gold bimetallic nanostructures with highly active surface-enhanced Raman scattering for specific detection and near-infrared photothermal therapy of human breast cancer cells. Anal Chem.

[CR71] Polson AG, Williams M, Gray AM (2010). Anti-CD22-MCC-DM1: an antibody−drug conjugate with a stable linker for the treatment of non-Hodgkin ’ s lymphoma. Leukemia.

[CR72] Rizzo A, Cusmai A, Acquafredda S (2022). Expert opinion on investigational drugs ladiratuzumab vedotin for metastatic triple negative cancer : preliminary results, key challenges, and clinical potential key challenges, and clinical potential. Expert Opin Investig Drugs.

[CR73] Roederer M (2001). Spectral compensation for flow cytometry: visualization artifacts, limitations, and caveats. Cytometry.

[CR74] Sanchez K, Kim I, Chun B (2021). Multiplex immunofluorescence to measure dynamic changes in tumor-infiltrating lymphocytes and PD-L1 in early-stage breast cancer. Breast Cancer Res.

[CR500] Schwenkert M, Birkholz K, Schwemmlein M, Kellner C, Nettelbeck DM, Schuler-Thurner B, Schaft N, Dörrie J, Kämpgen E, Fey GH (2008) The single-chain immunotoxin MCSP-ETA’, targeting melanoma-associated chondroitin sulfate proteoglycan, is a potent inducer of apoptosis in cultured human melanoma cells. Melanoma Res 18(2):73–84. 10.1097/CMR.0b013e3282f7c8f9 10.1097/CMR.0b013e3282f7c8f9 PMC2741307 18337643

[CR75] Shefet-Carasso L, Benhar I (2015). Antibody-targeted drugs and drug resistance—challenges and solutions. Drug Resist Updates.

[CR76] Smith LM, Nesterova A, Alley SC (2006). Potent cytotoxicity of an auristatin-containing antibody−drug conjugate targeting melanoma cells expressing melanotransferrin/p97. Mol Cancer Ther.

[CR77] Sommer A, Kopitz C, Schatz CA (2016). Preclinical efficacy of the auristatin-based antibody−drug conjugate BAY 1187982 for the treatment of FGFR2-positive solid tumors. Cancer Res.

[CR78] Sørlie T, Perou CM, Tibshirani R (2001). Gene expression patterns of breast carcinomas distinguish tumor subclasses with clinical implications. Proc Natl Acad Sci USA.

[CR79] Strebhardt K, Ullrich A (2008). Paul Ehrlich’s magic bullet concept: 100 years of progress. Nat Rev Cancer.

[CR80] Szalóki G, Goda K (2015). Compensation in multicolor flow cytometry. Cytom Part A.

[CR81] Tang H, Liu Y, Yu Z (2019). The analysis of key factors related to ADCS structural design. Front Pharmacol.

[CR82] Theunissen JW, Cai AG, Bhatti MM (2018). Treating tissue factor-positive cancers with antibody−drug conjugates that do not affect blood clotting. Mol Cancer Ther.

[CR83] Thomas P, Smart T (2005). HEK293 cell line: a vehicle for the expression of recombinant proteins. J Pharmacol Toxicol Methods.

[CR84] Touil Y, Zuliani T, Wolowczuk I (2013). The PI3K/AKT signaling pathway controls the quiescence of the low-rhodamine123-retention cell compartment enriched for melanoma stem cell activity. Stem Cells.

[CR85] Uranowska K, Kalic T, Valtsanidis V (2021). Expression of chondroitin sulfate proteoglycan 4 (CSPG4) in melanoma cells is downregulated upon inhibition of BRAF. Oncol Rep.

[CR86] von Felbert V, Bauerschlag D, Maass N (2016). A specific photoimmunotheranostics agent to detect and eliminate skin cancer cells expressing EGFR. J Cancer Res Clin Oncol.

[CR87] Wahby S, Fashoyin-Aje L, Osgood CL (2021). FDA approval summary: accelerated approval of sacituzumab govitecan-hziy for third-line treatment of metastatic triple-negative breast cancer. Clin Cancer Res.

[CR08] Wang Y, Ikeda DM, Narasimhan B, Longacre TA, Bleicher RJ, Pal S, Jackman RJ, Jeffrey SS (2008). Estrogen receptor–negative invasive breast cancer: imaging features of tumors with and without human epidermal growth factor receptor type 2 overexpression. Radiology, 246(2):367–375. 10.1148/radiol.2462070169 10.1148/radiol.2462070169 18180338

[CR89] Wang X, Osada T, Wang Y (2010). CSPG4 protein as a new target for the antibody-based immunotherapy of triple-negative breast cancer. J Natl Cancer Inst.

[CR90] Wang X, Wang Y, Yu L (2010). CSPG4 in cancer: multiple roles. Curr Mol Med.

[CR91] Wang J, Browne L, Slapetova I (2021). Multiplexed immunofluorescence identifies high stromal CD68+PD-L1+ macrophages as a predictor of improved survival in triple negative breast cancer. Sci Rep.

[CR92] Woitok M, Klose D, Niesen J (2016). The efficient elimination of solid tumor cells by EGFR-specific and HER2-specific scFv-SNAP fusion proteins conjugated to benzylguanine-modified auristatin F. Cancer Lett.

[CR93] Woitok M, Klose D, Di Fiore S (2017). Comparison of a mouse and a novel human scFv-SNAP-auristatin F drug conjugate with potent activity against EGFR-overexpressing human solid tumor cells. Onco Targets Ther.

[CR94] World Health Organization (2021) Breast cancer. Available online: https://www.who.int/news-room/fact-sheets/detail/breast-cancer

[CR95] Yang J, Price MA, Neudauer CL (2004). Melanoma chondroitin sulfate proteoglycan enhances FAK and ERK activation by distinct mechanisms. J Cell Biol.

[CR96] Yang J, Price MA, Gui YL (2009). Melanoma proteoglycan modifies gene expression to stimulate tumor cell motility, growth, and epithelial-to-mesenchymal transition. Cancer Res.

[CR97] Yersal O, Barutca S (2014). Biological subtypes of breast cancer: prognostic and therapeutic implications. World J Clin Oncol.

[CR98] Zhao S, Zuo WJ, Shao ZM, Jiang YZ (2020). Molecular subtypes and precision treatment of triple-negative breast cancer. Ann Transl Med.

