# Supplementary material for: CSPG4 as a target for the specific killing of triple-negative breast cancer cells by a recombinant SNAP-tag-based antibody-auristatin F drug conjugate
Source: J Cancer Res Clin Oncol. 2023 Jul 11;149(13):12203–25. doi: 10.1007/s00432-023-05031-3 (PMC10465649; doi:10.1007/s00432-023-05031-3)
Supplement: Supplementary file 1 — Supplementary file1 (DOCX 526 KB) [file 432_2023_5031_MOESM1_ESM.docx]

**Supplementary Material**

**CSPG4 as a Target for the Specific Killing of Triple-Negative Breast Cancer Cells by a recombinant SNAP tag-based Antibody-Auristatin F Drug Conjugate**

Neelakshi Mungra ^1,2^, Fleury A. N. Biteghe ^3^, Zaria Malindi ^1,4^, Allan M. Huysamen ^5^, Maryam Karaan ^1^, Natasha S. Hardcastle ^1^, Rubina Bunjun ^6,7^, Shivan Chetty ^8^, Krupa Naran ^1^, Dirk Lang ^9^, Wolfgang Richter ^10^, Roger Hunter ^5^ and Stefan Barth ^1,11,*^

^1^Medical Biotechnology and Immunotherapy Research Unit, Department of Integrative Biomedical Sciences, Institute of Infectious Disease and Molecular Medicine, Faculty of Health Sciences, University of Cape Town, Cape Town 7700, South Africa; krnmar009@myuct.ac.za (M.K.); natasha.hardcastle@gmail.com (N.S.H.); krupa.naran@gmail.com (K.N.)

^2^Centre for Immunity and Immunotherapies, Seattle Children’s Research Institute, Washington 98101, United States of America; neelakshi.mungra@seattlechildrens.org (N.M.)

^3^Department of Radiation Oncology and Biomedical Sciences, Cedars-Sinai Medical, Los Angeles, United States of America; fleurinhi@gmail.com (F.A.N.B.)

^4^Laser Research Centre, Faculty of Health Sciences, University of Johannesburg, Doornfontein, Johannesburg 2028, South Africa; zaria.malindi@gmail.com (Z.M.)

^5^Department of Chemistry, PD Hahn Building, University of Cape Town, Cape Town 7700, South Africa; allan.huysamen@alumni.uct.ac.za (A.M.H.); roger.hunter@uct.ac.za (R.H.)

^6^Institute of Infectious Disease and Molecular Medicine, Department of Pathology, University of Cape Town, Cape Town 7700, South Africa; ruby.bunjun@uct.ac.za (R.B.)

^7^ Division of Medical Virology, Department of Pathology, University of Cape Town, Cape Town 7700, South Africa

^8^ School of Clinical Medicine, Faculty of Health Sciences, University of Witwatersrand, Braamfontein, Johannesburg 2000, South Africa; shivan.chetty@witshealth.co.za (S.C.)

^9^ Division of Physiological Sciences, Department of Human Biology, University of Cape Town, Cape Town 7700, South Africa; dirk.lang@uct.ac.za (D.L.)

^10^TUBE Pharmaceuticals, Wien 1110, Austria; wrichter@tubepharma.at (W.R.)

^11^South African Research Chair in Cancer Biotechnology, Department of Integrative Biomedical Sciences, Faculty of Health Sciences, University of Cape Town, Cape Town 7700, South Africa; stefan.barth@uct.ac.za (S.B.)

*****Correspondence: stefan.barth@uct.ac.za (S.B.); Tel.: +27-21-406-6938


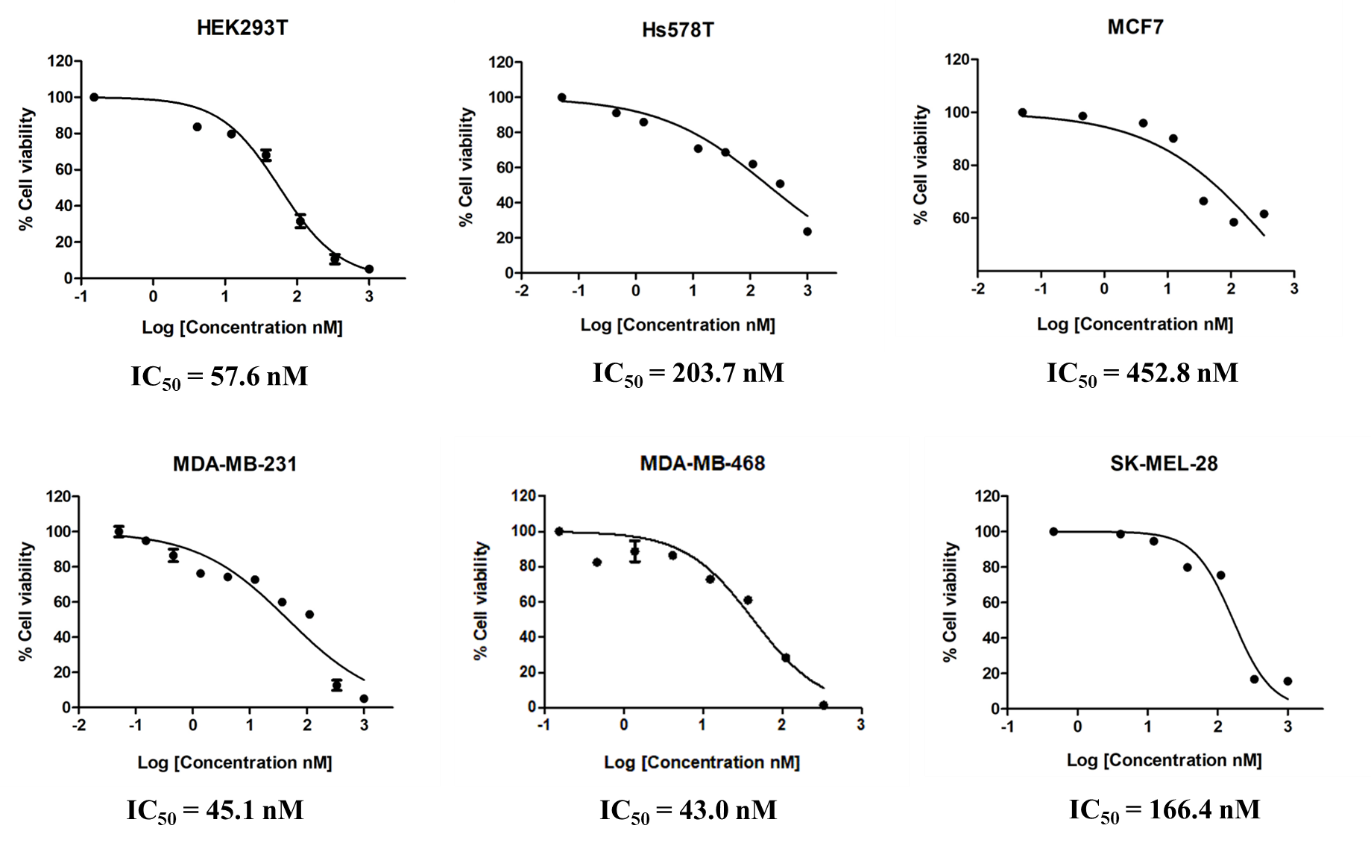


**Fig. S1** Dose-response curves demonstrating the cytotoxic activity of commercially sourced (unmodified) MMAF. The cytotoxic activity was assessed using an XTT-based viability assay after incubation with the drug for 72 hours. Cells were treated with (3-fold serially diluted) increasing concentrations of the drug and the IC_50_ values (relative to the untreated and Zeocin-treated (100 µg/ml) cells) were calculated using GraphPad Prism v5. Data are mean ± standard deviation (SD) of each measurement (presented as a percentage of cell viability), and the measurements were performed in triplicate at least 3 times

**
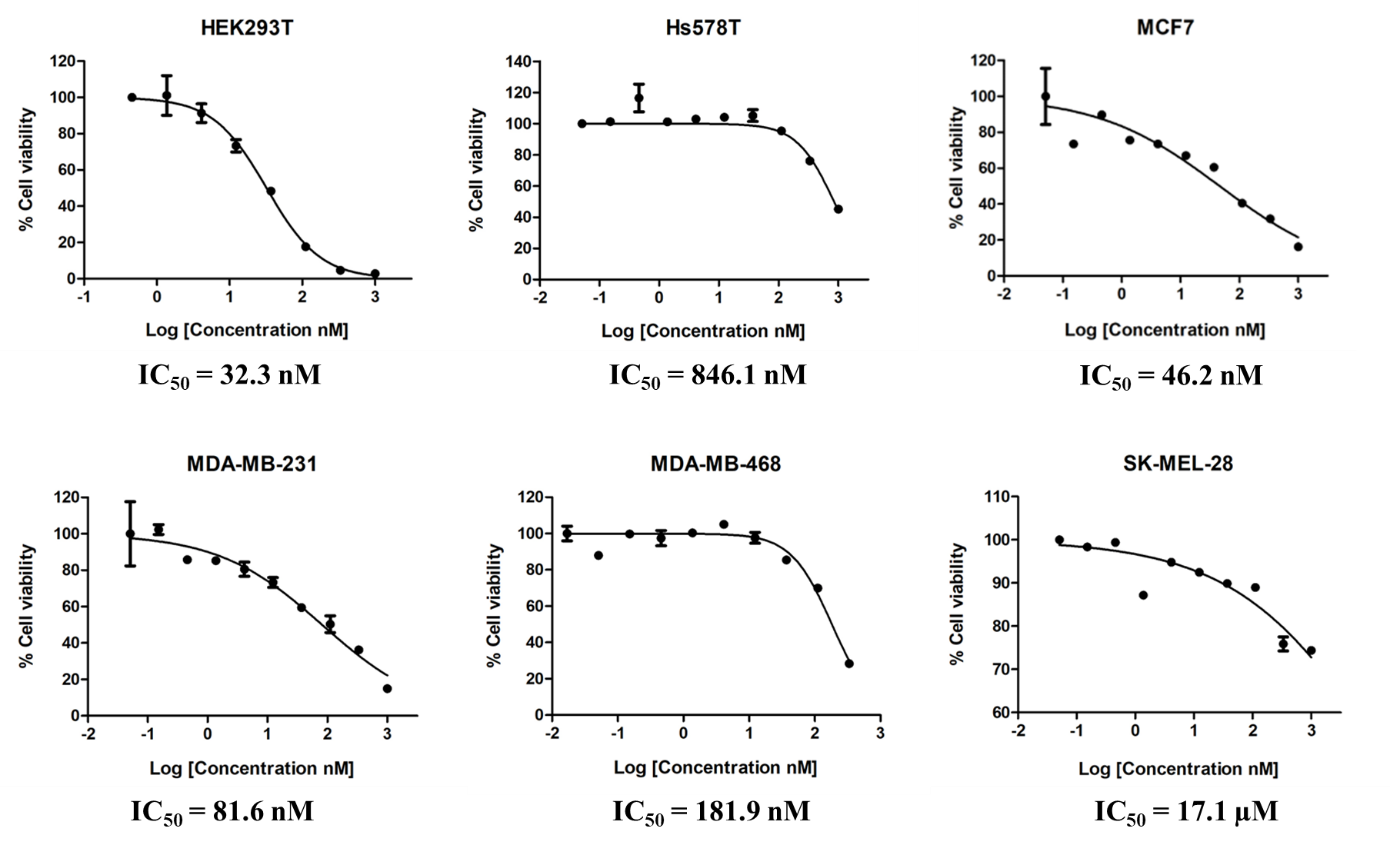
**

**Fig. S2** Dose-response curves demonstrating the cytotoxic activity of BG-modified AURIF. The cytotoxic activity was assessed using an XTT-based viability assay after incubation with the drug for 72 hours. Cells were treated with (3-fold serially diluted) increasing concentrations of the drug and the IC_50_ values (relative to the untreated and Zeocin-treated (100 µg/ml) cells) were calculated using GraphPad Prism v5. Data are mean ± standard deviation (SD) of each measurement (presented as a percentage of cell viability), and the measurements were performed in triplicate at least 3 times


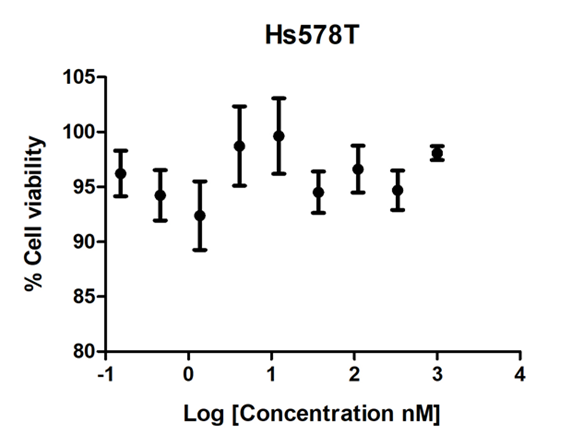


**Fig. S3** Dose-response curve demonstrating the negligible cytotoxic activity of the isotype control αASPH(scFv)-SNAP-AURIF on CSPG4^+^ Hs578T cells *in vitro*. The cytotoxic activity was assessed using an XTT-based viability assay after incubation with the drug for 72 hours. Cells were treated with (3-fold serially diluted) increasing concentrations of the drug and the IC_50_ values (relative to the untreated and Zeocin-treated (100 µg/ml) cells) were calculated using GraphPad Prism v5. Data are mean ± standard deviation (SD) of each measurement (presented as a percentage of cell viability), and the measurements were performed in triplicate at least 3 times
